# Peroxisomal cholesterol metabolism regulates yap-signaling, which maintains intestinal epithelial barrier function and is altered in Crohn’s disease

**DOI:** 10.1038/s41419-024-06925-x

**Published:** 2024-07-28

**Authors:** Marinella Pinelli, Stephanie Makdissi, Michal Scur, Brendon D. Parsons, Kristi Baker, Anthony Otley, Brad MacIntyre, Huong D. Nguyen, Peter K. Kim, Andrew W. Stadnyk, Francesca Di Cara

**Affiliations:** 1 Department of Microbiology and Immunology, Dalhousie University, Halifax, NS Canada; 2 Department of Pediatrics, Dalhousie University, Izaak Walton Killam (IWK) Health Centre, Halifax, NS Canada; 3 Department of Oncology, University of Alberta, Edmonton, AB Canada; 4 Department of Pathology, Dalhousie University, Halifax, NS Canada; 5 The Hospital for Sick Children, Department of Biochemistry, University of Toronto, Toronto, ON Canada

**Keywords:** Cell biology, Medical research

## Abstract

Intestinal epithelial cells line the luminal surface to establish the intestinal barrier, where the cells play essential roles in the digestion of food, absorption of nutrients and water, protection from microbial infections, and maintaining symbiotic interactions with the commensal microbial populations. Maintaining and coordinating all these functions requires tight regulatory signaling, which is essential for intestinal homeostasis and organismal health. Dysfunction of intestinal epithelial cells, indeed, is linked to gastrointestinal disorders such as irritable bowel syndrome, inflammatory bowel disease, and gluten-related enteropathies. Emerging evidence suggests that peroxisome metabolic functions are crucial in maintaining intestinal epithelial cell functions and intestinal epithelium regeneration and, therefore, homeostasis. Here, we investigated the molecular mechanisms by which peroxisome metabolism impacts enteric health using the fruit fly *Drosophila melanogaster* and murine model organisms and clinical samples. We show that peroxisomes control cellular cholesterol, which in turn regulates the conserved yes-associated protein-signaling and contributes to intestinal epithelial structure and epithelial barrier function. Moreover, analysis of intestinal organoid cultures derived from biopsies of patients affected by Crohn’s Disease revealed that the dysregulation of peroxisome number, excessive cellular cholesterol, and inhibition of Yap-signaling are markers of disease and could be novel diagnostic and/or therapeutic targets for treating Crohn’s Disease. Our studies provided mechanistic insights on peroxisomal signaling in intestinal epithelial cell functions and identified cholesterol as a novel metabolic regulator of yes-associated protein-signaling in tissue homeostasis.

## Introduction

Inflammatory bowel diseases (IBD) encompass two main idiopathic pathologies, ulcerative colitis (UC) and Crohn’s disease (CD), chronic inflammatory disorders of the digestive tract involving intestinal epithelial homeostasis and functions. Both diseases are characterized by relapsing-remitting inflammation of the colon and/or rectum (UC) or any part of the digestive tract (CD) as a result of abnormal innate and adaptive immune responses, changes in luminal and mucosa-associated microbiota, and alterations in epithelial function [1]. Lining of the digestive system is a single layer of epithelial cells consisting of a variety of highly specialized epithelial cell sub-types with a complete turnover period of 24 to 96 h [2]. The proliferative compartment of the epithelium is localized at the bottom of an intestinal crypt, followed by a gradient of increasingly differentiated epithelial cells along the vertical axis [3, 4].

The intestinal epithelium is more than just a physical barrier responsible for nutrient uptake; rather, it plays a critical role in homeostasis through its intrinsic, innate immune functions and maintains, under ideal circumstances, mutualistic and symbiotic relationships with a diverse and dynamic community of microorganisms and the host [5]. Genetic and environmental factors (e.g., diet) can cause alteration of signaling that leads to dysfunctional intestinal epithelial cells (IEC), which have been linked to IBD pathogenesis [6, 7]. IBD patients often display systemic metabolic changes concomitantly with increased inflammatory parameters [8–12], and studies of animal models of IBD further support these findings [13–16]. Specifically, an impairment in lipid metabolism is a typical metabolic signature associated with IBD that results in an alteration of intestinal barrier function [17]. Metabolomic studies in IBD patients identified lipid metabolites that can discriminate the patients from healthy controls [18]. Although the number of studies in patients is limited, animal studies revealed that chronic inflammation is associated with lipid metabolic dysfunction, leading to substantial cellular organelle damage in the intestinal epithelium, which exacerbates metabolic alteration in IEC and affects their functions [19–25]. Yet the underlying mechanism behind the lipid dysregulation remains unclear.

Peroxisomes are small, ubiquitous organelles present in virtually all eukaryotic cells. Peroxisomes are indispensable for many metabolic functions [26–28], including lipid metabolism, such as fatty acids α- and β-oxidation, ether phospholipid synthesis, cholesterol homeostasis, and primary bile acids synthesis [29]. The crucial role of peroxisomes in human health is unveiled in the Peroxisome Biogenesis Disorders (PBDs), a group of rare severe diseases caused by pathogenic variants of genes known as *Peroxins* (*Pex*) genes. To date, more than 30 *Pex* genes have been identified in various species. *Pex* genes encode proteins, which are fundamental for peroxisome biogenesis and functions [30]. Patients affected by PBDs can present sequelae of conditions including hypotonia, craniofacial malformations, various neurological deficits, and multi-organ failure [31, 32]. Some patients exhibit gastrointestinal bleeding [33], suggesting that peroxisome function is necessary to maintaining intestinal homeostasis. Moreover, peroxisomes are highly enriched in the gut [34, 35], and our group previously reported that a *Drosophila melanogaster* model that lacks functional peroxisomes in the IEC exhibits signs of intestinal dysplasia with an accumulation of progenitor cells (enteroblasts), production of inflammatory cytokines by IEC, dysregulation of the immune pathways, increased oxidative stress and IEC cell death [36]. These data suggest that peroxisomes are important regulators of IEC health and that altered signaling due to peroxisomes affects intestinal homeostasis and possibly leads to intestinal diseases such as IBD.

Here, we present molecular mechanisms that directly link peroxisome to homeostasis of the intestinal epithelium. Using a multi-organismal approach that includes a *Drosophila melanogaster* model with dysfunctional peroxisomes in IEC [36], a severe PBD murine model [37], mouse and human intestinal organoids (MIO and HIO, respectively), and organoid-derived monolayers, we discovered that dysfunctional peroxisomes affect intestinal epithelial barrier structure and function. Our findings demonstrate that dysfunction in peroxisome activities in IEC leads to cholesterol accumulation in IEC, affecting the yes-associated protein (Yap) signaling pathway. The Hippo-Yap pathway is a highly evolutionarily conserved pathway that regulates organ size by controlling cell proliferation, cell growth, stem cell renewal, and apoptosis [38–40]. The pathway is important in maintaining intestinal homeostasis, and in the past few years, studies have revealed its importance in gastrointestinal tract physiology and gastrointestinal diseases, including colorectal cancer and IBD [41, 42].

This study uncovers a novel role for peroxisomal lipid metabolism in regulating the Hippo-Yap pathway to maintain the structure and function of the intestinal epithelium, adding new material to the growing body of knowledge about peroxisome biology and Yap-signaling. Moreover, our work indicates that dysregulation of peroxisome metabolisms and Yap-signaling could be markers for intestinal inflammatory pathologies such as CD and paves the way for future research into developing therapeutic approaches aimed at peroxisome regulation of IEC function for treating intestinal inflammatory diseases.

## Materials and methods

### Ethics approval and consent to participate

Mouse colonies were maintained as stable inbred lines in the Swiss Webster and 129SVEV backgrounds under the institutionally approved animal protocol #21-023, abiding by the standards of the Canadian Council on Animal Care.

The research involving human material is in accordance with the Declaration of Helsinki.

Duodenal biopsies from patients and healthy donors were collected under the approval of IWK Health REB number #1024364.

Informed consent was obtained by all subjects.

### *Pex2* mutant mice

The *Pex2* mutant mouse strain 129S6.129-*Pex2tm1Plf*/Mmmh (null allele) [37] was provided by the Mutant Mouse Resource & Research Centers (MMRRC) supported by the National Institutes of Health. The homozygous null mutants in the congenic strain 129S6.129-*Pex2tm1Plf*^+/−^ showed variable embryonic lethality. Approximately 20% of homozygous null mutants survived to birth but were hypotonic, did not feed, and died on the day of birth. Homozygous null mutant mice that survived into the postnatal period were obtained by mating congenic 129S6.129-*Pex2tm1Plf*^+/−^ mice with wild-type Swiss Webster strain mice. F1-*Pxmp3tm1Plf*^*+/−*^ hybrids (designated Sw129) were then intercrossed to obtain Sw129-*Pxmp3tm1Plf*^*−/−*^ (indicated in the text as *Pex2*^*−/−*^) mice. The controls used were *Sw129-Pxmp3tm1Plf*^*+/+*^ (indicated in the text as WT).

### Fly stocks and husbandry

Pex5 dsRNA expressing lines *w*^*-*^; PattP40; *P{GD14972}v42332* and *w*^*1118*^ were from the Vienna *Drosophila* Resource Center (VDRC). The midgut-specific driver, Mex-GAL4, line *w*^*1118*^; *P{mex1-GAL4.2.1}* [43] was a gift from Kirst King-Jones, University of Alberta. The *magro*-expressing line *w*^*1118*^; *P{w[+mC]* *=* *UAS-mag.S}2* was from the Bloomington Drosophila Stock Center (BDSC). Pex5 cDNA flies were generated in our laboratory. Pex5 cDNA was cloned in the pTW Gateway vector and inserted on Chromosome 3 to generate the transgenic flies (*Pex5pTW* indicated in the panels as Pex5^OE^) using the Rainbow Transgenic Flies service.

Male and female 20-day-old adult flies were used in each experiment performed in this work.

Flies were maintained at 25 °C on standard BDSC cornmeal medium; Files that were fed an high fat diet (HFD) were kept on CM food for a week and then moved for 24 h (h) on CM supplied with 15% coconut oil (Sigma–Aldrich) and 5% erucic acid (Sigma–Aldrich).

### FFPE slides

Newborn pups were sacrificed at postnatal day 0, and small and large intestinal tissues were fixed in 10% buffered formalin for 24 h at 4 °C. Samples were then processed in a Leica ASP6025 Tissue Processor. Briefly, samples were dehydrated in increasing concentrations of ethanol (70-80-95-100%), then treated with xylene and finally embedded in paraffin into metal molds to create paraffin blocks. Blocks were cut at 5 µm slides at microtome and stained with the automated system Tissue Tek Prisma Stainer (Sakura), then coverslipped with the Tissue Tek glass coverslipper (Leica, Wetzlar Germany). We used at least three mice from different litters of each genotype. Images were collected using an Axio Observer Microscope with an Axiocam 105 color camera (Zeiss, Oberkochen, Germany) and analyzed with ImageJ software version 10 [44].

### Immunofluorescence on cryosections

Newborn pups were sacrificed at postnatal day 0, and the intestinal tissues were put in cryomolds in a solution made of 2/3 Tissue-Tek O.C.T. Compound (Sakura Finetek, Torrance, CA) and 1/3 20% sucrose in 1x Phosphate Buffered Saline (PBS). Samples were left to equilibrate for 1 h at room temperature (RT), then frozen in liquid nitrogen and stored at −80 °C. The frozen blocks were cut and fixed in acetone for 13 min, then left to air-dry overnight. The following day, sections were rehydrated in PBS for 5 min and then incubated then incubated in a blocking solution of 5% Fetal Bovine Serum (FBS) in PBS plus 0.01% Tween20 (Sigma–Aldrich) for 1 h at RT. Primary antibodies were prepared in blocking solution plus 0.01% Tween 20 and incubated overnight at 4 °C in a humidified chamber. The sections were washed three times in PBS and incubated for 1 h at RT with the appropriate secondary antibody, prepared in a blocking solution. After five washes in PBS, samples were mounted with ProLong Gold Antifade Mountant (Thermo Fisher Scientific, Waltham, MA, USA). Images were collected using a 20X (NA = 0.5) or a 63X (NA = 1.4) oil immersion objective mounted onto an Axio ObserverM1 Microscope and analyzed with ImageJ or Imaris 9 software by Bitplane. All the antibodies were used at the dilution recommended by the manufacturer and are reported in Table 1 in Supplementary data.

**Table 1:** List of used antibodies.

Target	Source	Identifier
Chromogranin A	Bioss	BS-0539R
E-cadherin	Abcam	ab11512
Ki-67	Invitrogen	53-5698-82
Claudin 7	Invitrogen	34-9100
Desmoplakin	PROGEN	61024
Histone H3 (phospho S10)	Abcam	ab14955
Active Caspase3	BD pharmingen	559565
Olfm4	Cell signaling technology	39141
Sucrase-Isomaltase	Santa cruz biotechnology	sc-393424
Yap	Cell signaling technology	14074
phospho-Yap	Cell signaling technology	4911
Phospho-Mst1/2	Cell signaling technology	3681
Phospho-Hpo	Cell signaling technology	49332
CD49f	BD pharmingen	555734
Coracle	DSHB	C566.9
Shotgun (E-cadherin)	DSHB	DCAD2
Discs large 1	DSHB	4F3 anti-discs large-c
Armadillo	DSHB	N2 7A1
Alexa fluor™ 555 Donkey anti-Mouse	Invitrogen	A31570
Alexa Fluor™ 488 Donkey anti-Rat IgG	Invitrogen	A21208
Alexa Fluor™ Donkey anti-Rabbit	Invitrogen	A-21206
Rabbit polyclonal anti SKL	Prof. Rachubinski, UAlberta	[45]
Cytokeratin 20	Agilent	M7019

### Mouse intestinal organoids culture

The protocol for crypt isolation is adapted from the one available at the STEMCELL Technologies (Vancouver, BC) website. All the solutions, except for the Intesticult Organoid Growth Medium (STEMCELL Technologies), were kept on ice and used cold. On day 0, the pups were sacrificed through decapitation, and the small and large intestines were harvested in cold sterile 1X PBS. After two washes with cold 1X PBS, the tissue was minced in the smallest pieces possible with sterile scissors inside the tube. The pieces were washed three more times and incubated with 10 mL of Gentle Cell Dissociation Reagent (STEMCELL Technologies) for 15 min at RT on a rocking platform. After washing with 1X PBS plus 0.1% BSA the fragments were passed through a 70 μm filter, and then washed again in DMEM/F-12 with 15 mM HEPES (STEMCELL Technologies). The crypts were resuspended in a cold solution made of ½ Growth Factor Reduced Matrigel (Corning Inc., Corning, NY, USA) and ½ DMEM/F-12 (STEMCELL Technologies) and seeded in 50 μL drops in a pre-warmed 24-well plate (Corning Inc.). The seeding concentration was 200 crypts/dome. The plate was incubated for 10 minutes at 37 °C to let the Matrigel polymerize, and then the pre-warmed complete Intesticult Organoid Growth Medium was added, 500 μL per well. Media changes were performed every two days, and organoids were passaged approximately once a week at a 1:4–1:6 ratio.

### Human intestinal organoids culture

Duodenal biopsies from patients and healthy donors were collected under the approval of IWK Health REB number #1024364. To generate and grow intestinal organoids, we used the Intesticult Organoid Growth Medium (Human) from STEMCELL Technologies and followed the manufacturer’s instructions to seed and passage them. Media changes were performed every two days, and organoids were passaged approximately once a week at a 1:4 ratio.

### Monolayer from mouse and human intestinal organoids

To generate a monolayer culture, MIOs were cultured for at least 1 passage with Intesticult OGM Human medium. Before seeding cells for monolayer, Transwell inserts (6.5 mm, 0.4 μm pore size, polyester, Corning Inc.) were coated with diluted Matrigel (1:50 in PBS) for 3–4 h. After removing the media, the organoids were incubated with TryPLE Express (Gibco, Billings, MT, USA) for 5 min at 37 °C and passed through an 18-gauge needle to obtain a single-cell suspension. The suspension was centrifuged, resuspended in Intesticult OGM Human medium (for mouse monolayers) or Intesticult ODM Human (for human) plus 10 mM Y-27632 (STEMCELL Technologies) and seeded at a concentration of 200,000 cells/insert. The following day, the medium containing unattached cells was removed and replaced with a fresh medium. A complete medium change was performed every other day until the monolayer reached confluence. In each analysis, we established four monolayers from four mice per genotype and/or condition.

### Immunofluorescence on MIO

Intestinal organoids grown for 4–6 days after passaging were used for whole-mount staining. Organoids were fixed in cold 4% paraformaldehyde (PFA) in 1X PBS for 30 min on ice with shaking, then permeabilized in PBS with 1% Triton X-100 (PBT) for 30 min on ice. To reduce autofluorescence, organoids were incubated for 30 min at RT in a 10 mM glycine solution prepared in PBS. After blocking in 5% BSA in PBT, organoids were incubated overnight at 4 °C with the primary antibody. The organoids were then washed three times with 0.2% Triton X-100, 0.05% Tween 20 in 1X PBS, and stained with secondary antibodies for 90 min. The samples were washed five times and mounted with a drop of ProLong Gold Antifade Mountant (Invitrogen). Images were acquired using an LSM 880 Confocal Microscope (Zeiss) and analyzed with ImageJ.

### Immunofluorescence on organoid-derived monolayers

For immunofluorescence, confluent monolayers were fixed in 4% PFA for 10 min, then permeabilized in 0.1% PBT for 10 min and blocked in 5% BSA for 1 h. The membranes were cut from the inserts using a razor blade and incubated overnight with the appropriate primary antibody in a humidified chamber. After three washes in 0.05% Tween 20 in 1X PBS, the membranes were incubated with the appropriate secondary antibody for 1 h at RT, then washed again and mounted on a slide with a drop of ProLong Gold Antifade Mountant. For cholera toxin staining, MIO-derived monolayers were incubated with 10 µg/ml of Cholera Toxin Subunit B (Invitrogen) for two hours at 37 °C before fixation.

### Filipin III staining on MIO-derived monolayers

Confluent monolayers from mouse organoids were fixed for 10 min in 4% PFA and washed in 1X PBS. The membranes were cut with a razor blade and put face-down on a drop of 50 µg/mL Filipin III ready-made solution (Sigma–Aldrich), then incubated at RT in the dark for 2 h. The membranes were washed in PBS and mounted. Images were collected immediately to avoid photobleaching of Filipin III. For the fly intestines, after dissection in cold PBS, they were put in a cold fixing solution (4% PFA in 1X PBS) at 4 °C overnight on a rotating platform. The tissue was washed three times with PBS and incubated with 50 ug/mL Filipin III solution for 30 min. The intestines were then washed in PBS and mounted.

### RNA extraction

Mouse organoids and fly intestines were collected in 500 μL of TRIzol reagent (Thermo Fisher Scientific) and snap-frozen in liquid nitrogen. For mouse organoids, total RNA was extracted using the RNeasy Mini kit (Qiagen, Venlo, The Netherlands), following the protocol. For fly intestines, total RNA was extracted using the Direct-zol™ RNA MicroPrep Kit (Cedarlane, Burlington, ON), according to the manufacturer’s instructions. For RNA extraction from mouse intestines, small intestines from day 0 pups were dissected, washed in cold PBS, and snap-frozen in liquid nitrogen. RNA was extracted using the RNeasy Mini kit (Qiagen), following the manufacturer’s instructions.

### cDNA synthesis and quantitative real-time PCR

RNA was reverse transcribed using the High-Capacity cDNA Reverse Transcription Kit (Applied Biosystems, Waltham, MA, USA). cDNA was amplified using the PowerTrack SYBR Green Master Mix (Applied Biosystems) following the manufacturer’s instructions. The reactions were loaded on a QuantStudio 6 Flex real-time PCR system (Applied Biosystems). Primer sequences are listed in Table 2. In supplementary documents.

**Table 2:** List of used primers.

Target gene	Forward primer sequence	Reverse primer sequence
Lgr5	CCTACTCGAAGACTTACCCAGT	GCATTGGGGTGAATGATAGCA
Olfm4	AGTGACCTTGTGCCTGCC	CACGCCACCATGACTACA
Chga	ATGACAAAAGGGGACACCAA	GTCTCCAGACACTCAGGGCT
Vil1	ATCTCCCTGAGGGTGTGGAC	AGAGAAGGCAGCTGGAGTCA
Lyz1	GGATGGCTACCGTGGTGTCAAGC	TCCCATAGTCGGTGCTTCGGTC
Muc2	ACCTCCAGGTTCAACACCAG	GTTGGCCCTGTTGTGGTCT
Yap1	CCAGACGACTTCCTCAACAGTG	GCATCTCCTTCCAGTGTGCCAA
Acly	AGGAAGTGCCACCTCCAACAGT	CGCTCATCACAGATGCTGGTCA
Fasn	CACAGTGCTCAAAGGACATGCC	CACCAGGTGTAGTGCCTTCCTC
Insig1	ATAGCCACCATCTTCTCCTCCG	CCAACGAACACGGCAATACAGC
Scd1	GCAAGCTCTACACCTGCCTCTT	CGTGCCTTGTAAGTTCTGTGGC
Pex5	CACCTCTTTCC GCCCATTGGT	TTAATCCTTAAAGGCCTCATT

### Immunofluorescence on *Drosophila* gut

Whole guts were dissected in cold 1X PBS and immediately put in cold fixing solution (4% PFA in 1X PBS); samples were incubated in fixing solution at 4 °C overnight on a rotating platform. The tissue was washed three times with 0.1% PBT for 30 min and blocked for 1 h in 5% normal goat serum in PBT. The primary antibodies were diluted in a blocking solution and incubated overnight at 4 °C. After three washes, the guts were incubated with the secondary antibody and DAPI diluted in a blocking solution for 2 h at RT. The guts were then washed three times and mounted. Images were collected with an LSM 880 Confocal Microscope (Zeiss) and analyzed with ImageJ.

### Detection of apoptosis

In situ end labeling of DNA fragments (TUNEL) was performed on organoids, monolayers, and tissue cryosections using the In situ Cell Death Detection kit, Tetramethyl rhodamine (TMR) red (Roche, Basel, Switzerland), following the manufacturer’s instructions with some modifications. Briefly, the samples were fixed and permeabilized following the immunofluorescence protocol; the working solution was prepared by mixing the Enzyme Solution and the Label Solution with a 1:9 proportion and incubated for 30 min at 37 °C. Samples were washed, stained with DAPI, and mounted. Visualization was done using an LSM 880 Confocal Microscope and analyzed with ImageJ.

### Western blotting

MIO-derived monolayers or fly guts were lysed in cold Ephrussi–Beadle Ringer’s buffer diluted 1:2 in Laemmli Sample Buffer (Bio-Rad, Hercules, CA, USA) and supplemented with PhosSTOP (Roche), 10 mM DTT and protease inhibitor cocktail cOmplete (Roche); a volume of 50 µL was used for each 24-well insert. Samples were then sonicated and incubated for 10 min at 100 °C then stored at −80°. The protein concentration in the lysates was determined using the Qubit Protein Assay Kit (Thermo Fisher), according to the manufacturer’s instructions. The samples were run (15 µg) on a 10% SDS-PAGE gel. At the end of the electrophoresis, the proteins were transferred onto a nitrocellulose membrane, the membrane was blocked for 1 h in 5% BSA in TBST (150 mM NaCl, 20 mM Tris-HCl, pH 7.5, 0.05% Tween 20), then incubated overnight at 4 °C with primary antibody. The following day the membrane was washed in TBST and incubated with the secondary antibody for 1 h. After three washes, the membrane was developed with a ChemiDOC Imaging System (Bio-Rad), using the Clarity™ Western ECL Substrate (Bio-Rad), following the manufacturer’s instructions. Image quantification was done in ImageJ: briefly, rectangular ROI was created around the bands, and their intensities were measured. A threshold was set to select the area under the curve. The area value is the result.

### Permeability assay

Confluent monolayers were washed once with 1X PBS, then we placed 500 µl of HBSS was added to the lower chamber of the Transwells and 200 µL of 1 mg/mL FITC-Dextran (Sigma–Aldrich) in 1X Hank’s Balanced Salt Solution (HBSS) added to the upper chamber. After 10 h, the lower chamber was collected the fluorescence intensity read on a CLARIOstar Plus microplate reader (BMG Labtech, Ortenberg, Germany).

### Smurf assay

16/18-day-old female and male flies were moved from regular cornmeal food to vials containing a vial plug saturated with a solution of 5% sucrose in PBS plus blue food coloring. After 48 h the flies were imaged with a 12.4X magnification on a SteREO Discovery V20 Microscope, equipped with an Axiocam 208 color camera (Zeiss). Image analysis was performed in ImageJ. For MβCD treatment, we prepared cornmeal food containing 10 mM MβCD and fed the flies on it for 48 h before conducting the Smurf assay.

### Transmission electron microscopy (TEM)

Samples were fixed for 2 h in 2.5% glutaraldehyde in 0.1 M sodium cacodylate buffer. After rinsing in 0.1 M sodium cacodylate buffer, samples were fixed for 2 h in 1% osmium tetroxide, then rinsed in dH_2_O and placed in 0.25% uranyl acetate overnight at 4 °C. Samples were then dehydrated in a graduated series of acetone, infiltrated and embedded in epon-araldite resin, and cured at 60 °C for 48 h. Thin sections were cut using a Reichert – Jung Ultracut E Ultramicrotome with a diamond knife (approximately 100 nm thick) and placed on 300 mesh copper grids which were then stained as follows: 2% aqueous uranyl acetate for 10 min, dH_2_O rinse, lead citrate for 4 min, dH_2_O rinse and air dry. The samples were then viewed using a JEOL JEM 1230 Transmission Electron Microscope at 80 kV, images were captured using a Hamamatsu ORCA-HR digital camera.

### ImageJ analyses

All antibodies that detected broadly expressed proteins were quantified by measuring the mean fluorescence intensity, and we plotted the ratio MFI/area of interest (e.g., MFI/organoid area for Sucrase-isomaltase). For Chromogranin-A, Olfm4, and pH3 antibodies, the quantification was done by manually counting the number of positive-stained cells within the area of interest (e.g., organoid or crypt).

#### TMR apoptosis quantification

For TMR red staining on MIO, we used the ‘3D Object Counter’ ImageJ tool with the size filter min set to 50; for MIO-derived monolayers and gut cryosections, we used the ‘Analyze particles’ tool, with the size filter set to 0–100 square micron. The values were then divided by the area of interest.

#### Cell junction quantification

To quantify the fluorescence intensity only along the cell-cell junctions, we thresholded (Otsu) the images on the staining channel, then created a selection, added it to the ROI manager, and measured the MFI in the ROI.

#### Smurf assay

To quantify the intestinal epithelium permeability, we manually drew an ROI around the dark blue area on the flies’ abdomen and measured it. Then, we drew and measured an area around the entire abdomen; we normalized the size of the blue area against the whole abdomen size.

#### Filipin puncta analysis

The puncta size was evaluated in ImageJ by creating ROIs with the polygonal selection tool around the bright Filipin-positive accumulations and measuring them.

#### Cell size measurement

To quantify the cell size, we created ROIs around the single cells with the polygonal selection tool and measured them. The cell area was measured using the ‘Marker-controlled Watershed’ segmentation of the ‘MorphoLibJ’ plugin in ImageJ. The cells were manually selected, and the software created areas around these seeding points and along the adherence junction protein staining and calculated the size of these areas in μm^2^.

#### p-Yap-cholera toxin colocalization analysis

The quantification was done by creating ROIs around the single cells and running the ‘Colocalization Threshold’ plugin for ImageJ.

### Imaris analyses

#### Desmosome distribution analysis

The distribution of desmoplakin puncta in gut cryosections and MIO-derived monolayer was analyzed using Imaris 9.9. Using the Surfaces’ tool we manually drew regions of interest in the image areas where the puncta were clearly defined and in focus; then we used the ‘Spots’ tool on the red channel, setting ‘Estimated XY diameter’ to 0.300 µm and using the filter ‘Shortest Distance to Surfaces’ <0, to select only the spots inside the ROI. Finally, we measured the ‘Average Distance To 5 Nearest Neighbours’ for the cryosections and the ‘Average Distance To three Nearest Neighbours’ for the monolayers. The monolayers were never completely flat, so we could focus on smaller desmosome groups along single-cell membranes, while in the flat cryosections, we could see sharp puncta of many cells together, thus we decided to measure a slightly different parameter for the two analyses. We plotted the values as density plots using R (R Core Team (2022). R: A language and environment for statistical computing. R Foundation for Statistical Computing, Vienna, Austria. https://www.R-project.org/).

#### Quantification of SKL-positive puncta

For SKL staining analysis, we used the ‘Spots’ tool to select the red channel and set the size range to 0.1–1 µm. The spots were filtered using the filter ’Quality’ with the automatic threshold. The nuclei were manually counted, and the total spot number was divided by the nuclei number to have the spots/cell value.

### Statistical analysis

Most of the statistical analyses were performed in GraphPad Prism version 10.0.2, using a two-tailed Student’s *t*-test, one-way ANOVA, or two-way ANOVA to calculate statistical significance when comparing the mean of two groups, more groups to a chosen control mean, and more than two groups.

For the analysis of desmoplakin puncta distribution in mouse intestine cryosections and MIO-derived monolayers, we used R to perform a Kolmogorov-Smirnov test using the dgof package (https://CRAN.R-project.org/package=dgof).

## Results

### Peroxisome dysfunction affects villus size in the small intestine of a *Pex2*^*−/−*^ PBD mouse model

**Fig. 1:**
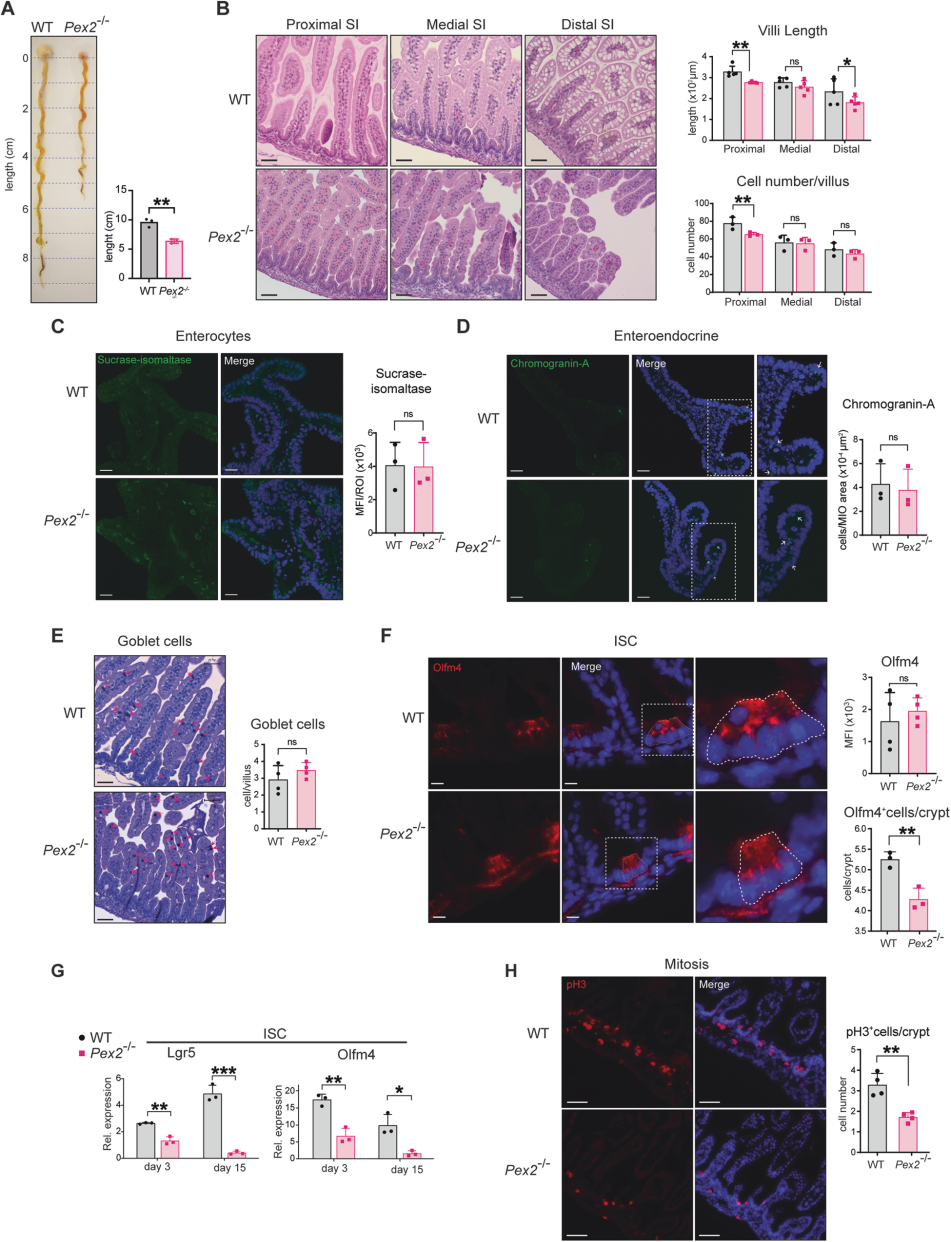
*Pex2* null allele affects villus length and ISC number in murine small intestines. **A** Representative image of dissected guts from WT and *Pex2*^*−/−*^ pups at postnatal day 0. The bar graph on the right represents the quantification in cm of the length of the intestinal tract in WT and *Pex2*^*−/−*^ pups at day 0. **B** Images of hematoxylin and eosin staining of small intestine sections from 3 WT and 3 *Pex2*^*−/−*^ pups at day 0 pups; the small intestine was divided into three parts roughly corresponding to the duodenum, jejunum, and ileum. Scalebar = 50 µm. We measure the length and cell number/villus of at least 50 villi of 3–5 mice for each genotype. **C** Representative image of IF microscopy of the enterocyte marker sucrase-isomaltase in MIO. The bar graph on the right represents the quantification of the mean fluorescence intensity (MFI) per organoid region of interest (ROI); *n* = 30 organoids developed from three mice. Scalebar = 20 µm. **D** Representative imaging of the enteroendocrine cells marker chromogranin A in MIO. The bar graph on the right reports the quantification of the number of Chromogranin A-positive cells per MIO area; *n* = 30 organoids developed from three mice. Scalebar = 20 µm. **E** Imaging of Alcian Blue/PAS (goblet cells) staining of formalin-fixed, paraffin-embedded intestinal tissue sections. Red arrows indicate goblet cells. The histogram reports the number of Alcian Blue/PAS-positive cells per villus; *n* = 220 villi from four mice for each genotype. **F** Imaging of Olfm4, a marker for ISC. The histograms report the MFI value per crypt (top) and the number of Olfm4-positive cells per crypt (bottom); *n* = 30 crypts from three different mice per genotype. Scalebar = 10 µm. **G** The bar graphs report the relative expression of *Lgr5* and *Olfm4* transcripts in WT and *Pex2*^*−/−*^ MIO at postnatal day 3 and day 15. Organoids were developed from *n* = 3 mice per genotype. **H** Imaging of pH3 in small intestine tissue cryosections. The bar graph represents the number of pH3-positive cells per crypt; *n* = 30 from three mice. scalebar = 50 µm. The error bars in all the graphs show the standard deviation, and significance was determined using Student’s *t*-test. ***p < 0.001; **p < 0.01; *p < 0.05; ns not significant.

To define the importance of peroxisome metabolism in maintaining intestinal epithelial health, we used a PBD murine model that carries a null mutation in the gene *Peroxin2* (*Pex2*^*−/−*^), which encodes for a ubiquitin ligase essential for peroxisome biogenesis and functions [37, 45]. Homozygous *Pex2*^*−/−*^ pups are smaller and lighter than age-matched wild-type littermates at birth (postnatal day 0). The homozygous mutants do not thrive and die in the first postnatal days. We dissected the small intestine and colon from day 0 pups and observed that their intestines are considerably shortener than Wild-Type (WT) intestines dissected from age-matched pups (Fig. 1A). Analyses of small intestine sections from paraffine-embedded intestines stained with hematoxylin and eosin showed that the length of intestinal villi and the number of cells per villus were significantly reduced in the proximal small intestine of the *Pex2*^*−/−*^ mice (Fig. 1B). To determine if the loss of peroxisome function leads to any changes in the colonic tissues of *Pex2*^*−/−*^ mice, we imaged colon section of *Pex2*^*−/−*^ and WT mice by optical and transmission electron microscopy (TEM). We observed an increase in the colonic luminal content of *Pex2*^*−/−*^ mice compared with WT mice, which is compatible with more epithelial shedding or slower content transit time (Figs. S1A, S1B). Using a terminal deoxynucleotidyl transferase dUTP nick end labeling (TUNEL) of colonic sections, we observed a significant increase in the number of apoptotic cells in the colonic lumen of *Pex2*^*−/−*^ mice compared with the age-matched WT littermates (Fig. S1C). This increase in colonic cell death partly explained why the colon tissue was friable and difficult to prepare for histology in *Pex2*^*−/−*^ mice. Therefore, the colonic regions were not further analyzed in this study. Thus, the lack of functional peroxisomes affects villi structure in the small intestines and impacts epithelial cell survival in the colonic epithelium in *Pex2*^*−/−*^ mice, supporting the hypothesis that peroxisomal requirements in the murine intestine might be different in different regions of the intestinal tract [46] as suggested by a previous morphological study [35].

### *Pex2*^*−/−*^ mice exhibit cell growth defects and reduced stem cell number in small intestines

To investigate if the reduced villi size in the small intestine of *Pex2*^*−/−*^ mutant mice is due to a differentiation defect, we performed indirect immunofluorescence (IF) imaging using antibodies specific for markers of the distinct differentiated cell types of the murine small intestinal epithelium. We analyzed cryosections of small intestines or mouse intestinal organoids (MIO) generated from the small intestine of postnatal day 0 *Pex2*^*−/−*^ mutant mice and WT littermates. We analyzed MIO at days 4–5 of culture, an established time-point to obtain fully differentiated epithelium in WT MIO [47]. We observed no significant difference in the number of enterocytes between WT and *Pex2*^*−/−*^ MIO, as revealed by antibody labeling of sucrase-isomaltase, a marker of fully differentiated enterocytes (Fig. 1C). Similarly, the enteroendocrine cell marker chromogranin A showed a similar signal in WT and *Pex2*^*−/−*^ MIO (Fig. 1D). To visualize the goblet cells and the intestinal stem cells, we stained tissue sections of small intestines from day 0 WT and *Pex2*^*−/−*^ mice. We stained the paraffine sections with Alcian Blue/ Periodic Acid Schiff (PAS) to detect mucins, a marker for goblet cells. Brightfield microscopy analyses did not show a significant difference in the number of goblet cells in the small intestine of *Pex2*^*−/−*^mice compared with WT (Fig. 1E). We performed quantitative reverse transcription polymerase chain reaction (RT-qPCR) analysis to measure the expression levels of lineage-specific markers in MIO and confirmed that no difference was found in the gene expression level of the enterocyte marker *Villin1* (*Vil1*), the goblet cell-specific marker *Mucin2* (*Muc2*), the Paneth cell lysozyme marker *Lyz1*, or the mature enteroendocrine marker *Chromogranin A* gene (*Chga*) at day 3 of culture (Fig. S1D). We then probed small intestinal sections to measure if any changes were apparent in intestinal stem cell (ISC) numbers in *Pex2*^−/−^mice, by IF and confocal microscopy. We detected a significant decrease in the number of ISCs per crypt in the small intestine of *Pex2*^*−/−*^ mice compared with WT (Fig. 1F). This finding was confirmed by RT-qPCR analyses that showed reduced expression of the ISC markers, *Lgr5* and *Olfm4*, in *Pex2*^*−/−*^-derived MIO versus WT MIO (Fig. 1G). To assess whether the loss of ISCs in the mutant small intestines was due to mitotic defects in ISC, we performed IF experiments on *Pex2*^*−/−*^ and WT intestinal cryosections to measure the amount of phosphorylated Histone H3 (pH3). *Pex2*^*−/−*^ small intestines exhibited fewer pH3-positive cells per crypt than WT (Fig. 1H), in agreement with the reduced number of Olfm4^+^ stem cells observed in the *Pex2*^*−/−*^ small intestines. We then cultured fully differentiated MIO-derived monolayers, confirmed by the expression of chromogranin A and cytokeratin 20 (CK20), a marker for enteroendocrine cells and a pan-IEC stain, respectively, (Fig. S1E). We assessed mitotic defects in MIO-derived monolayers using the Ki67 marker [48] as a proliferation marker because P-H3 did not work on organoids or MIO-derived monolayers in our hands. IF experiments showed a reduction in Ki67 signal in *Pex2*^*−/−*^ monolayers versus WT MIO-derived (Fi. S1F), suggesting that mitosis defects observed in *Pex2*^*−/−*^ small intestines are cell-autonomous and not caused by changes in extrinsic signaling.

Together, these analyses show that lack of functional peroxisomes does not affect differentiation of the intestinal epithelium but negatively impacts ISC number per crypt in *Pex2*^*−/−*^ small intestines compared with WT.

### Cells in the small intestine of a *Pex2*^*−/−*^ PBD murine model do not exhibit a high apoptotic rate

**Fig. 2:**
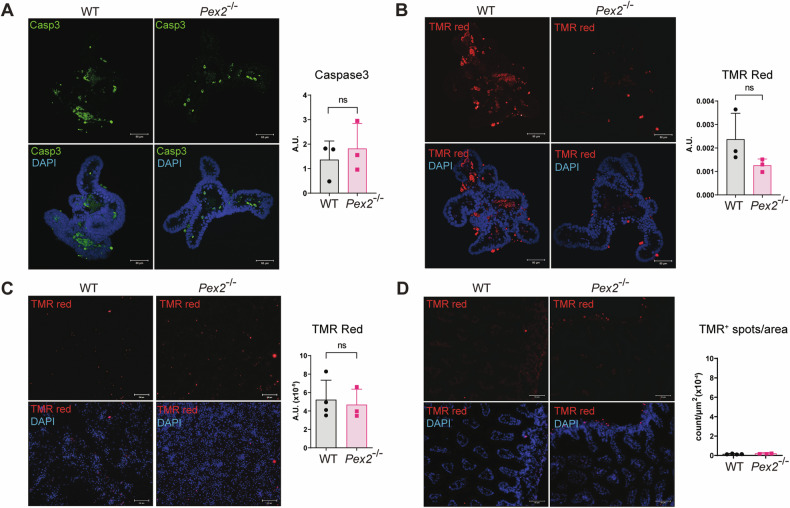
*Pex2*^*−/−*^ mice do not show increased cell death in the small intestine. **A** IF image of active caspase3 in Mio derived from WT and *Pex2*^*−/−*^ small intestines. The bar graph represents the Mean Fluorescence Intensity (MFI) on the sum projection of fluorescence per region of interest (ROI). *n* = 30 organoids from three mice per genotype. Scalebar = 50 µm. **B** Image of TUNEL-positive cells in Mio derived from WT and *Pex2*^*−/−*^ small intestines. The bar graph represents the arbitrary values of red positive-puncta per ROI. *n* = 30 organoids from three mice per genotype. Scalebar = 50 µm. **C** Detection of TUNEL-positive cells in MIO-derived monolayers. The bar graph represents the quantification of the number of TUNEL-positive dots in the total area reported in arbitrary units (A.U.). *n* = 30 monolayers derived from MIO from three mice per genotype. Scalebar = 200 µm. **D** Detection of TUNEL-positive cells in cryosections of the small intestine from WT and *Pex2*^*−/−*^ pups. *n* = 30 total sections derived from three mice per genotype. Scalebar = 50 µm. In all the graphs, the error bars represent the standard deviation. Significance was determined using Student’s *t*-test. ns not significant.

The observed reduction in villi size and cell number per villi in the *Pex2*^*−/−*^ small intestines could be the result of increased cell death. To test this, we performed IF and confocal microscopic analyses of MIO stained with an antibody specific for the cleaved and active form of caspase 3 (Casp3), a marker of apoptosis [49]. We did not observe any significant difference in Casp3-positive staining between WT and *Pex2*^*−/−*^ organoids (Fig. 2A). We repeated the analysis to probe for apoptosis in MIO using TUNEL staining and confirmed the results obtained for Casp3 staining (Fig. 2B). Confocal microscopy analyses of monolayers stained with TUNEL. revealed no difference in the number of TUNEL-positive cells between WT and *Pex2*^*−/−*^ intestinal monolayers (Fig. 2C). Cryosections of the small intestines were also stained using TUNEL. This analysis confirmed that WT and mutant samples have comparable apoptotic rates (Fig. 2D). This indicates that the changes in the small intestinal epithelium of mice lacking functional peroxisomes are not associated with an increase in apoptotic rates.

### Peroxisomes are required to maintain intestinal epithelial junctional integrity

**Fig. 3:**
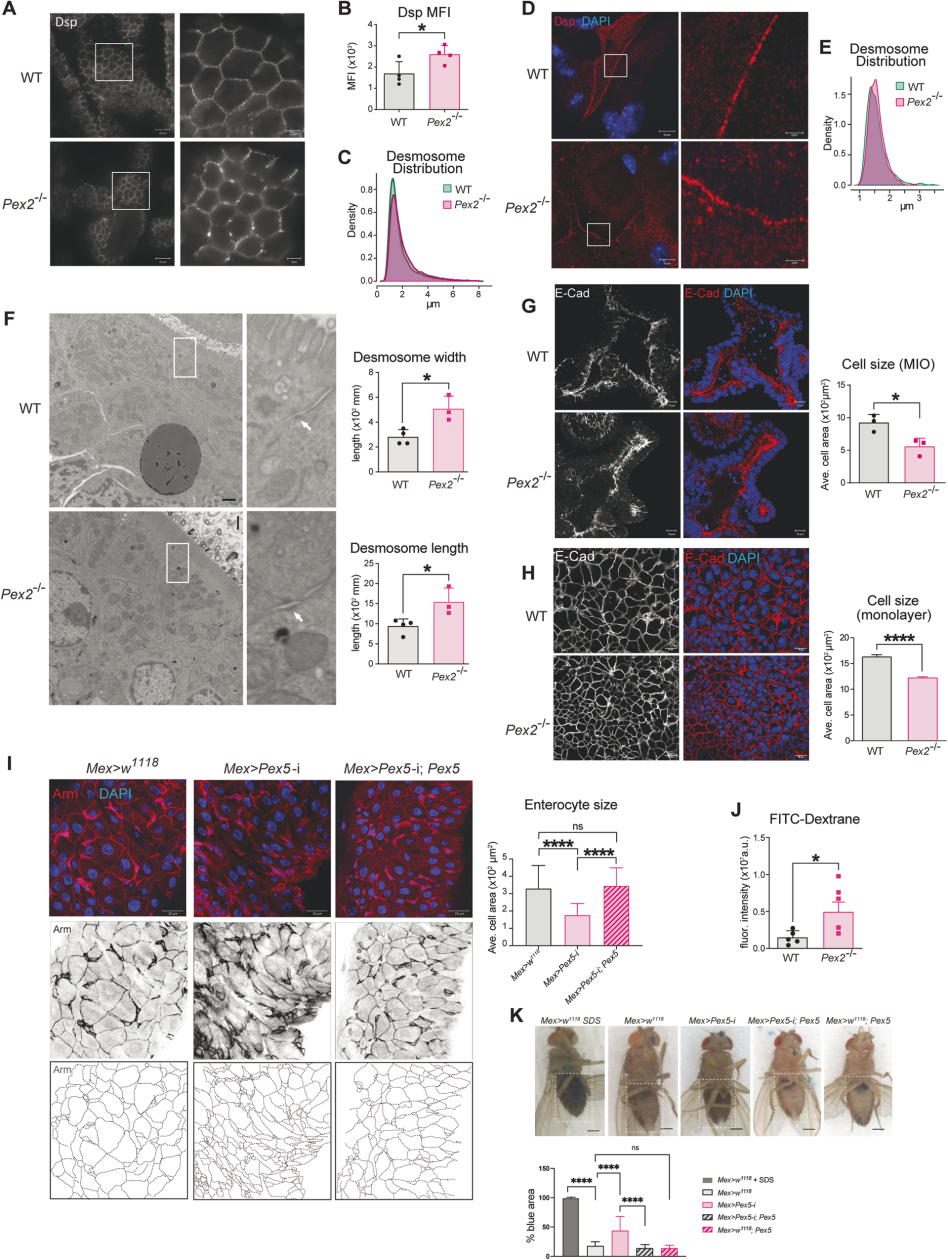
Intestinal epithelial cells in the Pex2^−/−^ small intestines show defects in desmosome distribution and reduction in cell size. **A** IF image of Desmoplakin (Dsp) in small intestine cryosections. *n* = 40 sections derived from four mice per genotype Scalebar = 20 µm. **B** The bar graph reports the values of MFI per image in WT and *Pex2*^*−/−*^ cryosections. **C** Desmoplakin puncta distribution is shown as a density plot histogram of the average distance measured in Imaris between the five nearest neighbors. The density plot histogram was created in R, and a Two-sample Kolmogorov-Smirnov test was run: *p*-value < 2.2e-^16^. **D** Imaging of Desmoplakin in WT and *Pex2*^*−/−*^MIO-derived monolayers. *n* = 40 monolayers derived from four mice per genotype Scalebar = 20 µm. **E** The average distance between the three nearest neighbors measured in Imaris was reported in a relative density plot histogram. Statistical significance was determined in a two-sample Kolmogorov-Smirnov test: *p*-value = 1.439e^-05^. **F** TEM imaging of small intestine sections from small intestines at day 0 pups, with desmosomes highlighted by the white arrows. Scalebar = 1 µm, 10,000X magnification. The length and thickness of desmosomes were manually measured in ImageJ, *n* = 3 from three mice per genotype. **G** Detection of E-cadherin in WT and *Pex2*^*−/−*^ MIO. Scalebar = 10 µm. The bar graph represents the cell size reported in μm^2^. *n* = 40 from four mice per genotype. **H** Imaging of E-cadherin in MIO-derived monolayers. *n* = 30 monolayers derived from three mice per genotype. Scalebar = 20 µm. The bar graph represents the average cell area reported in μm^2^. **I** Detection of Armadillo (Arm) in *Drosophila* intestines dissected from *Mex* > *w*^*1118*^, *Mex* *>* *Pex5*-i and *Mex* *>* *Pex5*-i; *Pex5*. *n* = 10 guts from 10 flies per genotype. The dotted lines in the right panels are enterocyte boundary traces of the above panels to illustrate enterocyte size. Scalebar = 10 µm. The bar graph represents the average cell area reported in μm^2^. **J** The bar graph represents the quantification of fluorescence intensity of FITC-dextran in permeability assays of MIO-derived monolayers; *n* = 5 monolayers. **K** Representative picture of the Smurf assay on 20-day-old female flies. The dotted lines in the right panels are enterocyte boundary traces of the above panels to illustrate enterocyte size. The bar graph indicates the percentage of dark blue area divided by the total abdomen area; scale bar = 500 µm, *n* = 20 flies per genotype and condition. In all the bar graphs, the error bars represent the standard deviation. Significance was determined using one-way ANOVA in **I** and **K** and Student’s *t*-test for all the other graphs. ****p < 0.0001; **p < 0.01; *p < 0.05; ns not significant.

To better define the structure of the intestinal epithelium and the villi in *Pex2*^*−/−*^ small intestines, we performed IF on cryosections of small intestines dissected from postnatal day 0 pups using antibodies against cell junction proteins. We stained for claudin7 [25], E-cadherin [50], and Cd49f [51], markers of tight junctions, adherens junctions, and hemidesmosomes, respectively (Fig. S1G, H, I). Indirect IF experiments did not show significant differences in staining or distribution of any of the three markers between controls and mutants. We also performed IF experiments on tissue stained with desmoplakin, a marker of desmosomes [52] since desmosomes are essential to maintain intestinal epithelial structure and function in health and disease [52]. We detected a significant increase in desmoplakin staining in the mutants compared with the WT (Fig. 3A, B). Moreover, desmoplakin exhibited a unique distribution along the cell-cell contact line in the homozygous *Pex2*^−/−^ small intestine compared with the WT. Measurements of the average distance between desmoplakin puncta validated that desmoplakin and, therefore, desmosomes had an increased and more variable distance in the intestinal epithelium of *Pex2*^*−/−*^ pups, as represented by the density plot histogram (Fig. 3C). We confirmed the result when we analyzed the desmosomes distribution in MIO-derived monolayers from *Pex2*^*−/−*^ pups compared with WT (Fig. 3D, E). TEM imaging of murine small intestine sections also indicated an abnormal desmosome structure (arrowheads point at intermediate filaments organized at desmosome junctions) [53] in *Pex2* mutants, with an increase in the intercellular space, in both desmosome length and width (Fig. 3F). Indirect IF and confocal microscopy analyses of small intestines stained with desmoplakin also suggested that the cell size of the intestinal epithelium might be reduced in the mutant compared with the WT (Fig. 3A).

To determine whether cell size was reduced in *Pex2*^*−/−*^ intestinal epithelial cells in the small intestine, we measured the cell size in MIO and in MIO-derived monolayers stained with E-Cad, which strongly highlights the cell boundaries. We used MIO and MIO-derived monolayers also to determine whether the defects in cell size were caused indirectly by developmental and growth delays in the *Pex2*^*−/−*^ pups or directly by cell-autonomous alteration of growth signaling caused by peroxisomal dysfunction in the IEC. We observed a significant decrease in the cell size in *Pex2*^*−/−*^ intestines in both ex vivo models, significantly smaller than cells in WT MIO and MIO-derived monolayers (Fig. 3G, H). These results confirm that *Pex2*^*−/−*^ mice has intestinal cell growth defects, which are intrinsic to the IEC.

To further confirm that the cell size defect we observed in the intestines of *Pex2*^*−/−*^ mice was caused by a tissue-specific requirement for peroxisomes in controlling cell growth and not by global growth defects in *Pex2*^*−/−*^ mice, we analyzed the cell size of EIC in guts of the *Drosophila melanogaster* that lack functional peroxisomes exclusively in IEC (also referred to as enterocytes in *Drosophila*) [36]. This *Drosophila* model is generated by RNA interference (RNAi)-mediated depletion of *Peroxin5* (Pex5), a gene that encodes for a protein that is essential for peroxisome biogenesis and function and the stability of which is affected in *Pex2*^*−/−*^ mutants in mammals [45]. The *Pex2* gene encodes a ubiquitin ligase needed to recycle the *Pex5* receptor, therefore cells that have a depletion or ablation of Pex5 show peroxisome biogenesis defects similar to those observed in *Pex2* mutant mice, such as a loss of targeting of peroxisomal enzymes to peroxisomes [36]. Because the commercially available RNA-i inducible Pex2 *Drosophila* strains do not trigger an efficient depletion of Pex2 transcript and protein, we used the *Pex5* RNAi strain for our studies in the *Drosophila* model. We previously validated the Pex5 depletion efficiency and described this model (*Mex* *>* *Pex5*-i) [36] and reported that their intestinal epithelia show dysplasia, alteration of core cellular growth and metabolic signaling, and constitutively express inflammatory cytokines compared to age-matched controls (*Mex* > *w*^*1118*^) [36]. *Mex* *>* *Pex5*-i flies do not show growth defects compared to age-matched *Mex* > *w*^*1118*^ control flies. We performed IF analyses on dissected intestines using antibodies specific for the cell adhesion protein β-Catenin (adherence junction), also known as Armadillo (Arm) [54]. Confocal microscopic analyses of the stained fly midguts and measurement of the enterocytes (cells with larger nuclei) size in the fly midguts (small intestine) showed that *Mex* *>* *Pex5*-i flies had significantly smaller cells, confirming that peroxisomes are required for controlling cell size in IEC in *Drosophila* as in the mouse (Fig. 3I). We also observed an increase in Arm staining in progenitor cells (cells with small nuclei) of *Mex* *>* *Pex5*-i, which indicates higher Wnt pathway activation in the mutant compared with control; this is linked to increased progenitor cells and epithelial dysplasia [55] that we previously described in *Mex* *>* *Pex5*-i intestines [36]. Overexpression of wild-type Pex5 cDNA in *Mex* *>* *Pex5*-i enterocytes rescues cell size and progenitor number to the level observed in control midguts (Fig. 3I).

To define whether dysfunctional peroxisomes in the IEC cause defects in cell junction in the *Mex* *>* *Pex5*-i *Drosophila* model, we performed indirect IF and confocal analyses to detect other cell junction proteins in intestines of *Mex* *>* *Pex5*-i flies and age-matched controls. We used antibodies against Coracle (Cora) for septate junction, Discs large 1 (DLG1) for basolateral junction, and DE-Cadherin (DE-cad) for adherence junction (Fig. S2A–C) [52, 56]. Of note, *Drosophila* epithelial cells do not have desmosomes, but they have adherens and septate junctions (functionally similar to tight junctions in mammals) [57]. The distribution of each marker did not appear different in *Mex* *>* *Pex5*-i midguts compared with control midguts. The quantification of these markers showed no difference in septate junction or basolateral junction markers; however, we observed an increase in the staining of the adherence junction in progenitor cells, confirming an increase in progenitor cells in *Mex* *>* *Pex5*-i flies (Fig. S2C) [58] as we previously reported [36], but also suggesting alteration of cell junctions that correlate with an increase of DE-cad staining intensity and distribution [59]. Thus, the lack of functional peroxisomes in *Pex2*^*−/−*^ mutant small intestines in mice and *Mex* *>* *Pex5*-i midguts in *Drosophila melanogaster* causes cell size defects and alterations in adherens junctions, which might indicate dysregulated intestinal barrier integrity.

### Dysfunctional peroxisomes alter intestinal barrier function

Defects in tight junctions, adherence junctions, and desmosomes cause alteration in intestinal barrier integrity and play a distinct role in IBD pathogenesis. Since dysfunctional peroxisomes cause abnormal expression and/or distribution of cell junctional markers in the small intestine of *Pex2*^*−/−*^ mutant mice and adult intestines of *Mex* *>* *Pex5-i* flies, we tested whether the intestinal epithelial permeability of the intestines was affected in both model systems. Since the *Pex2*^*−/−*^ mutant mice die at day 1 to 3 post-birth in our experience, we could not assess intestinal epithelial permeability in vivo. Instead, we performed a FITC-dextran permeability assay on *Pex2*^*−/−*^ mutant and WT MIO-derived monolayers [60]. Primary intestinal cells from *Pex2*^*−/−*^ mutant and WT MIO were cultured to form a confluent monolayer growing on a microporous semipermeable membrane filter in a Transwell apparatus. We added FITC-dextran molecules into the upper compartment and replaced the culture medium from the lower compartment with Hanks′ Balanced Salt Solution (HSS). After 24 h, we measured the fluorescence of samples collected from the acceptor compartment of WT and *Pex2*^*−/−*^ mutant MIO-derived monolayers. We detected higher FITC fluorescence in the samples from the lower compartment of the *Pex2*^*−/−*^- derived monolayers compared with WT ones (Fig. 3J). The increase in the FITC-fluorescence signal in the lower compartment is directly proportional to the permeability of the cell monolayer and, therefore, indicates that the *Pex2*^*−/−*^ intestinal epithelium is compromised.

Considering our finding that *Pex2*^*−/−*^- derived monolayers demonstrate increased permeability, we predicted that intestinal permeability of the *Drosophila melanogaster Mex* *>* *Pex5-*i flies would also be increased. We used the Smurf assay [61] to assess the loss of intestinal barrier function in *Mex* *>* *Pex5*-i, in vivo. In the assay, the permeability is detected by the presence of a nonabsorbable blue food dye outside of the digestive tract after feeding, hereinafter referred to as Smurf [61, 62]. We assessed males and females *Mex* *>* *Pex5-i* and *Mex* > *w*^*1118*^. Both male and female *Mex* > *w*^*1118*^ flies showed the blue-colored food retained in the intestine *w* while *Mex* *>* *Pex5*-i flies showed blue dye throughout the abdomen. We reported only the data gathered from the analyses of the females because quantifying the blue area in the abdomen of males was challenging due to their smaller size compared to female flies. *Mex* > *w*^*1118*^ fed for 24 h with sucrose in 2% SDS solution to trigger epithelial barrier damage were used as a positive control for increased permeability and exhibited the blue dye throughout their body. In conclusion, *Mex* *>* *Pex5-*i were partially “Smurfs”, indicating that the barrier function of the intestinal epithelium was compromised in flies with dysfunctional peroxisomes in the IEC (Fig. 3K). Overexpression of wild-type Pex5 cDNA in *Mex* *>* *Pex5-*i rescued the “Smurfs” (Fig. 3K). Thus, alteration of peroxisomal function in IEC compromises intestinal epithelial barrier function in mice and *Drosophila*.

### Yap signaling is altered in intestines with dysfunctional peroxisomes

**Fig. 4:**
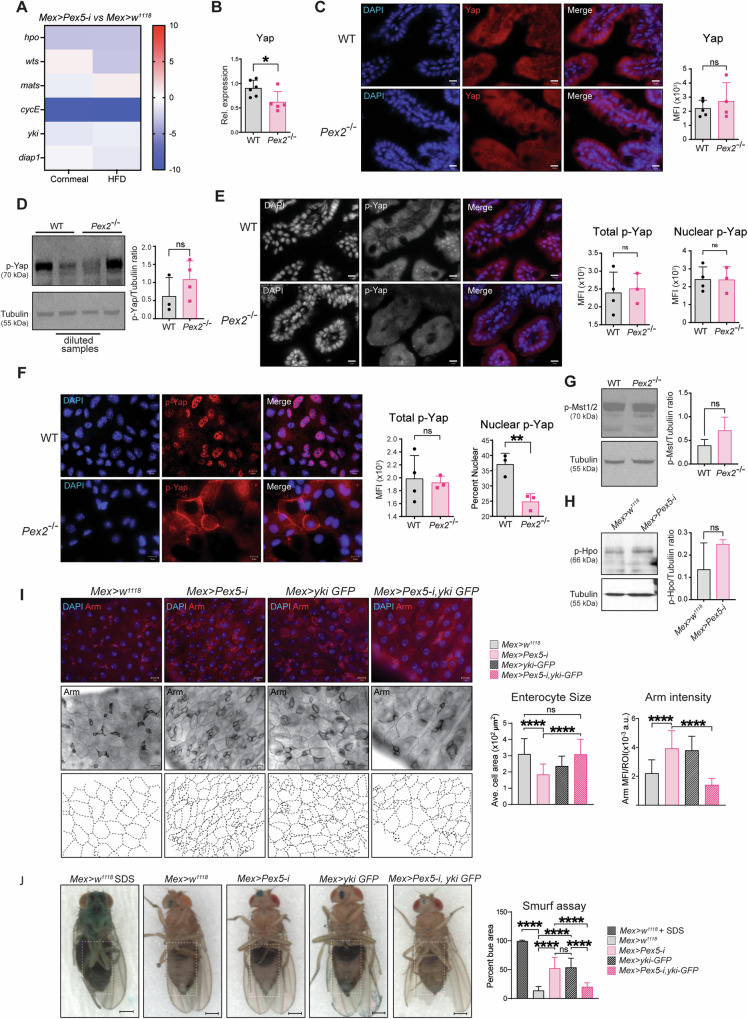
Yap/Yki signaling is reduced in *Pex2*^*−/−*^ and *Mex* *>* *Pex5-*i intestines and affects intestinal epithelial structure and function. **A** The heatmap represents the ratio of log2 fold change expression of genes encoding for proteins of the Hippo pathway in *Mex* *>* *Pex5-*i versus control flies as found in the RNA-seq screen on *Drosophila* intestines dissected from flies raised on regular cornmeal or high-fat diet (HFD). **B** The bar graph reports the amount of Yap transcript in WT and *Pex2*^*−/−*^ small intestines. *n* = 6 mice per each genotype. **C** Imaging of Yap in intestinal cryosections. The bar graph represents the MFI of Yap staining per ROI. The values reported were calculated on 10 images per experiment, *n* = 3 mice. Scalebar = 10 µm. **D** Western blot analyses of total protein extracts from MIO-derived monolayers to quantify p-YAP protein. The bar graph represents ratiometric analyses of the mean intensity value between p-Yap and α-Tubulin in western blots experiments. *n* = 3 mice. **E** IF image of p-Yap in small intestine cryosections. The bar graphs represent the MFI of p-Yap staining per ROI and the ratiometric analyses of the MFI between the p-Yap fluorescent signal and the DAPI fluorescent signal, respectively. The values reported were calculated on 10 images per experiment, *n* = 3 mice. Scalebar = 10 µm. **F** Imaging of p-Yap in MIO-derived monolayers. The bar graphs show the MFI of total p-Yap staining per ROI and the ratiometric analyses of the MFI between the p-Yap fluorescent signal and the DAPI fluorescent signal, respectively. The values reported were calculated on 10 images per experiment, *n* = 3 mice. Scalebar = 10 µm. **G** Western blot analyses of total protein extracts from MIO-derived monolayers to quantify p-MST1/2. The bar graph represents ratiometric analyses of the mean intensity value between p-Mst1/2 and a-Tubulin in western blots experiments. *n* = 3 extracts from MIO-derived monolayers from three mice. **H** Western blotting analyses of total protein extracts from *Drosophila* intestines of the reported genotypes. The bar graph represents ratiometric analyses of the mean intensity value between p-Hpo and α−Tubulin in western blots experiments. *n* = 25 guts. **I** IF image of Armadillo protein to detect progenitor cells (bright cells) and enterocyte boundaries (dim cells) in adult *Drosophila* guts; *n* = 12 guts. The dotted lines in the lower panels are enterocyte boundary traces of the above panels to illustrate enterocyte size. The bar graphs show the average cell area of enterocytes in μm^2^; *n* = 20 intestines per genotype Scalebar = 10 µm. J Representative picture of 20-days-old female flies of the reported genotypes fed with blue-colored food. The dotted lines in the right panels are enterocyte boundary traces of the above panels to illustrate enterocyte size. The bar graph indicates the percentage of dark blue area relative to the total abdomen area; scale bar = 500 µm, *n* = 20. In all histograms, the error bars represent standard deviations. Significance was determined using a one-way ANOVA test in **I** and **J** and Student’s *t*-test for all the other graphs. ****p < 0.0001; **p < 0.01; *p < 0.05; ns not significant.

To define the signaling by which peroxisomes control IEC structure and function, we performed an RNA-seq screen (Di Cara unpublished data) on intestines from *Mex* *>* *Pex5-i* flies and *Mex* > *w*^*1118*^ raised on a regular cornmeal diet (CM) or short-term high-fat diet (HFD, 15% coconut oil, and 5% broccoli oil) that is known to enhance intestinal permeability [63]. Differential gene expression analyses between *Mex* *>* *Pex5-*i intestines compared with the respective control in each feeding condition showed downregulation in several genes involved in the Hippo pathway (Fig. 4A). The Hippo pathway is an evolutionarily conserved pathway that is known to control cell growth and organ size and homeostasis through modulating cell proliferation, survival, apoptosis, and stemness [40]. The Hippo pathway is a highly conserved kinase cascade originally discovered in *Drosophila melanogaster* in genetic screens of tumor suppressors. The mammalian core kinases of the Hippo pathway consist of MST1/2 (ortholog of *Drosophila* Hippo/Hpo) [64], SAV1 (Salvador ortholog), LATS1/2 (Warts/Wts ortholog), and MOB1A/B (Mats ortholog). The transcriptional co-activators YAP (Yorkie/Yki ortholog) and TAZ (YAP paralog in mammals) are the primary downstream effectors of the Hippo pathway [65]. Phosphorylation of Yap/Yki results in retention of the protein in the cytoplasm, thus blocking its transcriptional activity [66, 67]. Since *Cyclin E* (*CycE*), an established target of Yki that controls cell proliferation in *Drosophila* tissues is one of the most significantly downregulated genes in *Mex* *>* *Pex5-*i compared with *Mex* > *w*^*1118*^ intestines, we hypothesized that peroxisomes regulate Yki activity. To verify whether Yki/Yap signaling was also dysregulated in *Pex2*^*−/−*^ intestines, we performed RT-qPCR for *Pex2*^−/−^ and WT-derived MIO to measure the amount of Yap transcript as a read-out of the pathway since the Yap expression level correlate with signaling activity in mammals [68–70]. Yap expression was significantly lower in *Pex2*^*−/−*^ MIO compared with WT (Fig. 4B). We then performed indirect IF to map Yap in cryosections of the small intestine. Median fluorescence intensity (MFI) of the protein amount per villi was not significantly different between WT and *Pex2*^*−/−*^ mutants (Fig. 4C). We also probed the amount and distribution of phosphorylated (inactive) Yap (p-Yap) in small intestines using western blotting and indirect IF, respectively. We found no difference in the amount of p-Yap in *Pex2*^*−/−*^ mutant small intestines compared with WT samples (Figs. 4D, S3). Interestingly, although western blotting analyses from MIO extract did not show a difference in p-Yap amount in Pex2^−/−^ compared with WT samples, we noticed that the p-Yap signal was diffuse in the cytoplasm and at the plasma membrane and not in the nuclei of *Pex2*^*−/−*^ cells in the small intestines. We performed indirect IF on WT and Pex2^−/−^ MIO-derived monolayers to further validate this localization in mutant tissue. Confocal microscopy imaging and analyses of the total and nuclear MFI for p-Yap indicate that the nuclear p-Yap was significantly reduced in *Pex2*^*−/−*^ samples (Fig. 4F), confirming that Yap activity is reduced in *Pex2*^*−/−*^ mutant small intestines. We probed whether the inhibition of Yap in *Pex2*^*−/−*^ mutant small intestines was caused by an increase in Mst1/2 (or Hpo in *Drosophila*) kinase signaling activation that, in turn, inhibits Yap/Yki [66]. To measure Mst1/2 activation, we checked for the level of phosphorylated threonine 183/180 of the Mst1/2 protein, respectively. We detected no difference in the phosphorylation of Mst1/2 when comparing *Pex2*^*−/−*^ MIO-derived monolayer lysates with WT lysates (Figs. 4G, S4). We also analyzed the phosphorylation status of Hpo in the *Drosophila Mex* *>* *Pex5-*i intestines and confirmed there was no difference in Hpo phosphorylation in the peroxisomal-depleted intestines compared to WT (Figs. 4H and S5).

We carried out genetic interaction studies to determine whether alteration of p-Yap localization caused defects in Yap-signaling and, in turn, in cell growth and epithelial barrier structure. We first tested whether the activation of Yki (ortholog of Yap in *Drosophila*) would rescue the defects observed in *Mex* > *Pex5-*i flies. To visualize cell junctions, we performed indirect IF using an antibody to stain the adherens junction protein Arm [71]. As reported above, we found that cells in *Mex* *>* *Pex5-*i midguts are significantly smaller than cells in the midgut of the age-matched relative control, *Mex* > *w*^*1118*^; however, when we overexpressed Yki [72] in enterocytes of *Mex* *>* *Pex5-*i midguts, the cell size was similar to cells in *Mex* > *w*^*1118*^ control intestines suggesting that defects in peroxisomal function may impede Yki-signaling and affect cell growth (Fig. 4I). We also noticed that the accumulation of cell clusters with strong Arm staining, which we reported in *Mex* *>* *Pex5-*i midguts was also rescued in *Mex* *>* *Pex5-*i; *yki* intestines. We then tested whether overexpression of Yki in enterocytes of *Mex* *>* *Pex5-*i midguts would rescue intestinal epithelial barrier function. We performed the Smurf assay and observed that while female *Mex* *>* *Pex5-*i flies showed again blue dye throughout the abdomen, the blue-colored food remained in the intestine of female *Mex* > *w*^*1118*^ flies and *Mex* *>* *Pex5-i*; *yki* flies (Fig. 4J). We conclude that activation of Yki signaling in *Mex* *>* *Pex5-*i restored epithelial barrier integrity. Noteworthy, the overexpression of Yki in the intestine also induced the flies to “Smurf”, possibly due to hyperactivation of Yki signaling in enterocytes inducing overgrowth and dysplasia [72], therefore affecting the epithelial barrier integrity. From the sum of our experiments, we conclude that dysfunctional peroxisomes cause the downregulation of Yap-signaling in mice and flies, which in turn affects cell growth, progenitor proliferation/differentiation, and ultimate intestinal epithelial barrier function.

### Accumulation of cellular cholesterol inhibits Yap-signaling

**Fig. 5:**
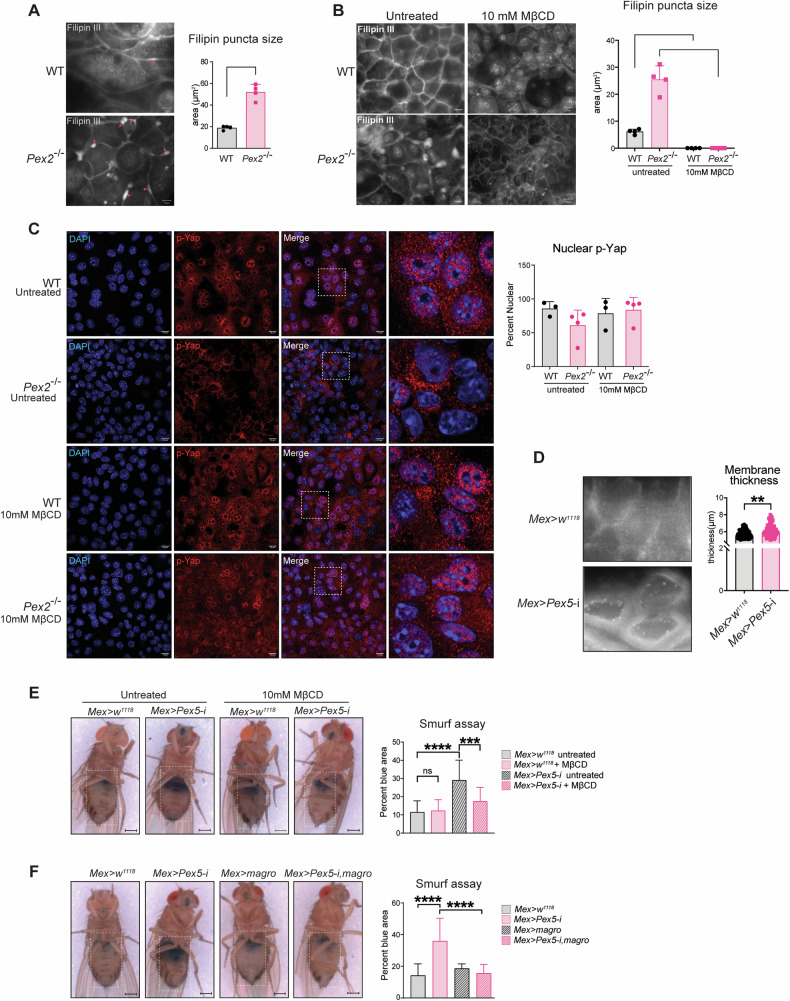
Dysfunction of peroxisomal metabolism alters cholesterol distribution in the cells and modifies Yap signaling. **A** Imaging of Filipin III staining to visualize cholesterol in WT and *Pex2*^*−/−*^ MIO-derived monolayers. The bar graph represents the size of Filipin III puncta (highlighted by the red arrows), *n* = 40 monolayers derived from four mice per genotype. Scalebar = 10 µm. **B** Immunofluorescence with Filipin III in WT and *Pex2*^*−/−*^ MIO-derived monolayer untreated and treated with 10 mM MβCD for 2 h. The bar graph represents the size of cholesterol puncta, *n* = 40 monolayers images derived from four established monolayers from four mice per genotype. Scalebar = 10 µm. **C** IF image of p-Yap in MIO-derived monolayer, untreated and treated with 10 mM MβCD for 2 h; The bar graph reports the percentage of p-Yap fluorescent signal that overlaps with the DAPI fluorescent signal. *n* = 30 monolayers derived from three established monolayers from three mice per genotype. **D** Imaging of FIlipin III staining in and *Mex* > *w*^*1118*^ and *Mex* *>* *Pex5-i* enterocytes; *n* = 20 intestines per genotype. Scalebar = 10 µm. **E** Representative pictures of *Mex* > *w*^*1118*^ and *Mex* *>* *Pex5*-i flies fed a regular cornmeal diet or a diet supplemented with 10 mM MβCD for 48 h before being tested in a Smurf assay. Scalebar = 500 µm. The dotted lines in the right panels are enterocyte boundary traces of the above panels to illustrate enterocyte size. The bar graph indicates the percentage of dark blue area relative to the total abdomen area; *n* = 20 guts per genotype and condition. **F** Representative pictures of flies of the reported genotypes tested in the Smurf assay. The dotted lines in the right panels are enterocyte boundary traces of the above panels to illustrate enterocyte size. The bar graph indicates the percentage of dark blue area in the total abdomen area; *n* = 20 guts per genotype. Scalebar = 500 µm. In all histograms, the error bars represent standard deviations. Significance was determined using Student’s *t*-test in **A** and **D** and a two-way ANOVA test for all the other graphs. ****p < 0.0001; ***p < 0.001; **p < 0.01; ns not significant.

Multiple studies have reported that metabolic signals influence Hippo-Yap signaling, which, in turn, controls genes that direct cellular and organismal metabolism [73, 74]. It has been reported that Yap can stimulate de novo lipogenesis and promote proliferation and tissue growth by activating the Sterol regulatory element binding proteins (SREBPs). SREBPs are a family of transcription factors that regulate lipid homeostasis by controlling the expression of a range of enzymes required for endogenous cholesterol, fatty acid, triacylglycerol, and phospholipid synthesis [75]. We probed the alteration of the SREBPs pathway in the *Pex2*^*−/−*^ small intestine. We performed RT-qPCR analyses to measure the expression of target genes of SREBPs. We did not find differences in the expression of any of the reported targets of the Yap-SREBPs in *Pex2*^*−/−*^ small intestines compared with WT (Fig. S3A), suggesting that this metabolic signaling was not responsible for the defects observed in the *Pex2*^*−/−*^ small intestine. Peroxisomes are essential for β-oxidation of VLCFs, α-oxidation of fatty acids, synthesis of ether lipids, and metabolism of cholesterol [76]. Peroxisomal lipid metabolism can affect cellular lipid signaling and membrane composition [77]. We hypothesized that alteration in peroxisomal lipid metabolism could cause alteration of membrane lipids and lipid domains essential to regulate cellular signaling [78], including Yap-signaling and the organization of cell junction proteins [79–81]. Cholesterol is an essential structural lipid in the plasma membrane and is an essential component of the lipid domains. Cholesterol modulates tight-junction and adherence junctions assembly/integrity [81–84] and was suggested to indirectly regulate Yki/Yap cellular localization [85] and signaling in cancer cells [79, 80]. Peroxisomes are critical for maintaining cholesterol homeostasis in the *Pex2*^*−/−*^ global mutants [86]; we probed for whether defects in cells of the murine *Pex2*^*−/−*^ small intestine and *Drosophila Mex* *>* *Pex5-*i midguts are caused by defects of cellular cholesterol accumulation or distribution. To visualize cholesterol, we stained MIO-derived monolayers with Filipin III, a fluorescent molecule that specifically binds to cholesterol in vivo. We observed the presence of numerous puncta along the cell membrane and in the cytoplasm that were significantly larger in *Pex2*^*−/*−^ samples (Fig. 5A), indicating defects in cholesterol distribution in mutant cells. To determine whether the accumulation of cholesterol in *Pex2*^*−/−*^ intestinal cells causes recruitment and/or alteration in Yap-signaling, we treated intestinal monolayer cultures with Methyl-β-cyclodextrin (MβCD), a drug with a high affinity for cholesterol that depletes cholesterol from membranes [87]. We performed a pilot dose-response experiment and found that treatment using 10 mM MβCD for 2 h depleted cholesterol in MIO-derived monolayers without affecting viability (Fig. S6B). When we treated the mutant monolayer with 10 mM MβCD for 2 h, we observed a depletion of cholesterol puncta from the *Pex2*^*−/*−^ monolayers (Fig. 5B), and p-Yap localization changed from membrane/cytoplasmic to the nucleus to a similar extend as observed in WT monolayers (Fig. 5C).

Peroxisome metabolism contributes to cholesterol synthesis and catabolism [76, 86]. To determine whether the accumulation of cholesterol at the plasma membrane in IEC was caused by defects in synthesis or catabolism of cholesterol, we stained midguts of *Mex* > *w*^*1118*^ and *Mex* *>* *Pex5-*i flies with Filipin III to detect the accumulation of cholesterol in intestinal cells in *Drosophila*. *Drosophila* is a cholesterol auxotroph organism [88]; therefore, any accumulation in cholesterol in *Mex* *>* *Pex5-i* intestines would likely be linked to catabolic pathway defects. Confocal microscopic analyses of Filipin III-stained midguts indicated an increase in Filipin III staining in *Mex* *>* *Pex5-*i enterocytes compared with sex and age-matched controls (Fig. 5D). To investigate if depletion of cholesterol by MβCD treatment could improve the defects in intestinal epithelial permeability of *Mex* *>* *Pex5-*i flies, we fed *Mex* *>* *Pex5-*i flies with the drug for 48 h, then performed the Smurf assay. The assay showed that the treatment led to a complete rescue of the permeability defects, and all the *Mex* *>* *Pex5-*i flies retained the blue-colored food in the intestine, similar to *Mex* > *w*^*1118*^ *control* flies, while untreated *Mex* *>* *Pex5-*i flies showed blue dye throughout the abdomen (Fig. 5E). We performed genetic interaction studies to confirm these data. We overexpressed the lipase *magro*, which encodes for intestinal cholesterol esterase required to hydrolyze cholesterol esters and promote cholesterol clearance [89] in enterocytes. Similarly to what was observed with MβCD treatments, overexpression of *magro* in *Mex* *>* *Pex5-*i midguts restored the epithelial barrier functions, as shown by the results of the Smurf assays (Fig. 5F). All together these data confirmed that peroxisomes regulate cellular cholesterol in enterocytes to modulate Yap/Yki signaling and control epithelial barrier function.

### Reduction in peroxisome number and p-Yap nuclear localization is observed in Human intestinal monolayer derived from IBD patients

**Fig. 6:**
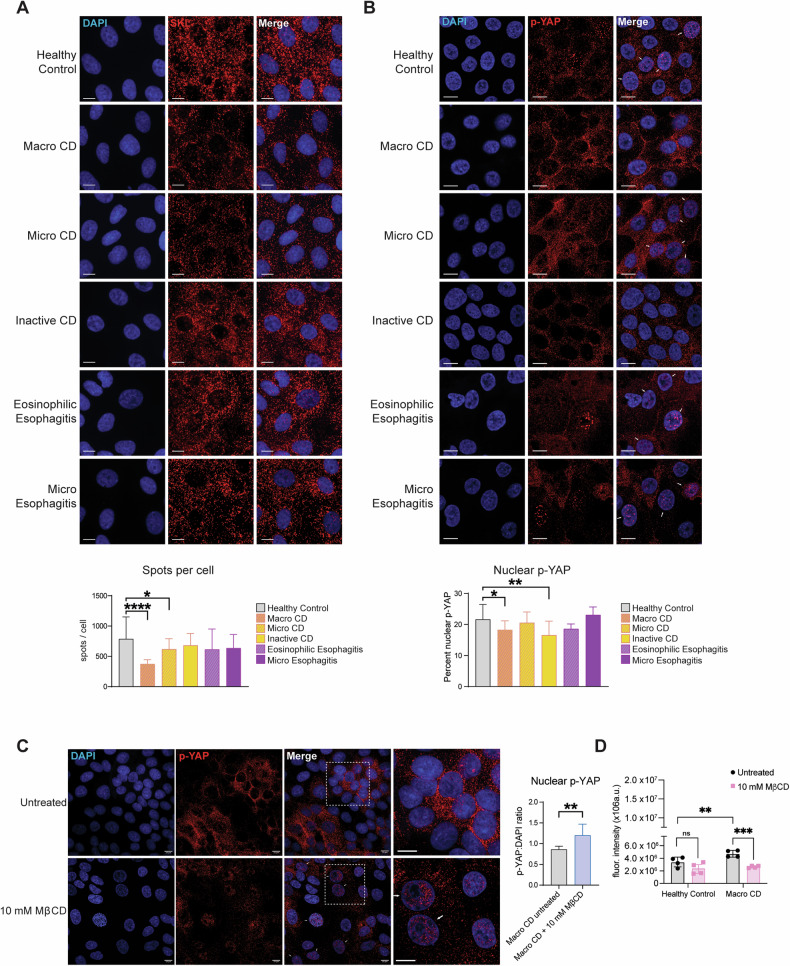
HIO-derived monolayers from IBD patients show peroxisome cholesterol-dependent recruitment of p-YAP out of the nuclei. **A** Detection of peroxisomes SKL staining in HIO-derived monolayers for the detection of peroxisomes. For patient samples *n* = 10 monolayers. For healthy controls, *n* = 30. Scalebar = 10 µm. The bar graph reports the number of SKL-positive spots per cell in each sample. **B** Detection of p-YAP in HIO-derived monolayers. For patient samples, *n* = 10. For healthy controls, *n* = 30. Scalebar = 10 µm. The bar graph reports the percentage of the p-YAP fluorescent signal overlapping with the DAPI fluorescent signal. **C** p-YAP staining in HIO-derived monolayer from the patient with macroscopic Crohn’s disease, with and without treatment with 10 mM MβCD for 2 h. Scalebar = 10 µm. The bar graph reports the amount of p-YAP fluorescent signal that overlaps with the DAPI fluorescent signal. **D** The bar graph represents the quantification of fluorescence intensity of FITC-dextran in permeability assays of HIO-derived monolayers; *n* = 4 monolayers from four patients and four healthy control intestinal biopsies. In all histograms, the error bars represent standard deviations. Significance was determined using one-way ANOVA in **A** and **B** and Student’s *t*-test in **C**. ****p < 0.0001; **p < 0.01; *p < 0.05; ns not significant.

We obtained endoscopic duodenal biopsies from pediatric patients affected by different inflammatory conditions of the gastrointestinal tract as well as patients ultimately determined not to have an intestinal inflammatory condition (controls) and generated human intestinal organoids (HIO) and HIO-derived monolayers from the biopsies. As peroxisome numbers change in inflammatory conditions [77, 90–92] and the lack of functional peroxisomes affects intestinal epithelial barrier function, we performed indirect IF of HIO-derived monolayers using an antibody against the peroxisomal targeting signal type 1 (PTS1) Serine-Lysine-Leucine-CO_2_H (SKL), a canonic marker for peroxisomes [93], to visualize peroxisomes numbers in IEC of patients and control donors. Confocal microscopic analyses showed a significant decrease in the number of peroxisomes per cell in the IEC of HIO derived from biopsies of patients affected by active macroscopic Crohn’s disease (CD) and, to a lesser though still significant extent, microscopic Crohn’s disease (CD) (Fig. 6A) compared with HIO derived from biopsies from control donors or of patients affected by eosinophilic esophagitis and micro esophagitis. We performed indirect IF to localize p-Yap in the HIO-derived monolayers from patients and control donors. We found a reduced fraction of nuclear p-YAP and an increased fraction of p-YAP at the plasma membrane and cytoplasm in active and inactive macroscopic CD intestines compared to the healthy controls (Fig. 6B). We also measured the amount of cholesterol in the membrane of active macroscopic CD patients and healthy controls by Filipin II staining and confocal microscopic analyses and found that membrane of IEC in monolayers of CD patients have higher Filipin III staining than what observed in the IEC in healthy control donors (Fig. S6C). We then treated the monolayer derived from active macroscopic CD patients with MβCD. We observed that the cholesterol scavenger drug resulted in greater p-YAP nuclear localization to a level similar to what was detected in the intestinal HIO monolayers derived from biopsies of control donors (Fig. 6C). Finally, we performed FITC-dextran permeability assay on monolayer derived from active macroscopic CD patients and IEC in healthy control donors [60]. Primary intestinal cells from macroscopic CD and healthy control donors were cultured to form a confluent monolayer growing on a microporous semipermeable membrane filter in a Transwell apparatus in the HIO differentiation medium without or with MβCD. We added FITC-dextran molecules into the upper compartment and replaced the culture medium from the lower compartment with HBSS. After 2 h, we measured the fluorescence of samples collected from the acceptor compartment of CD and healthy control HIO-derived monolayers. We detected higher FITC fluorescence in the samples from the lower compartment of the CD^-^derived monolayers than in the healthy control donors (Fig. 6D). The increase in the FITC-fluorescence signal in the lower compartment is directly proportional to the permeability of the cell monolayer and, therefore, indicates that the CD intestinal epithelium is compromised. However, FITC fluorescence in the samples from the lower compartment of MβCD-treated CD monolayer is comparable to FITC fluorescence in samples from lower compartments of MβCD-treated and untreated healthy control-derived monolayers (Fig. 6D).

Our results suggest that the IEC-specific dysregulation of peroxisomes and the consequent alteration of cellular cholesterol and inhibition of YAP-signaling are features of intestinal inflammatory diseases and compromised intestinal barrier function.

## Discussion

Previous research on peroxisomal function showed that the metabolic defects in peroxisome biogenesis disorders (PBDs) alter the development of multiple organs. For instance, peroxisomes are increasingly recognized to have a role in neural trophic survival and function in the brain, muscle function, and immune cell function, and are responsible for producing primary bile acids in liver hepatocytes [92, 94]. Yet, novel phenotypes continue to be described for PBDs, indicating that our understanding of the tissue-specific requirements for peroxisomes and how this supports organismal development and health is still incomplete. Some PBD patients exhibit gastrointestinal bleeding [33, 34], suggesting that peroxisomal function is needed to maintain intestinal epithelial barrier structure and functions. Since peroxisomes are particularly enriched and heterogeneous in the intestines of various organisms, including *Drosophila* [36], mouse [35], and humans [95]. This suggests that intestinal phenotypes of PBD may be due to alteration of tissue-specific functions for peroxisomes in the epithelium.

A microscopy study of the duodenal tissue of twenty-nine patients affected by various intestinal diseases found an alteration in peroxisome number, morphology, and cellular localization in the villi, indicating a correlation between peroxisomal function and intestinal pathology in humans [95]. In addition, a study that investigated the requirement for peroxisomes in ISC-mediated repair of the injured human intestine, found a significant increase of peroxisomes in both architecturally abnormal colon epithelia (both in crypts and villi, but more remarkably in crypts) of patients with Ulcerative colitis or Crohn’s Disease compared with the normal para-carcinoma region (used as controls) of patients with colorectal cancer [96]. To date, the molecular mechanisms by which peroxisomes contribute to maintaining intestinal epithelial homeostasis and function in health and disease are still not clear. In this study, using a multi-organismal approach, we demonstrated that dysfunctional peroxisomes affect cholesterol distribution in IEC and deregulate the Yap-signaling pathway, ultimately impacting the intestinal epithelial barrier structure and function in *Drosophila melanogaster*, mice, and humans.

Severe PBDs caused by global mutations of genes encoding peroxisomal biogenesis factors such as Pex2 lead to perinatal death in mice. While such mutations are an accurate mirror of the alteration in metabolic and genetic networks that lead to PBDs, they are challenging models to study the tissue-specific requirements for the organelle beyond development. Intestinal organoids and organoid-derived monolayers have emerged as a powerful model to study the complex cellular dynamics of the gut epithelium [47, 97]. Using intestinal organoids, we determined that dysfunctional peroxisomes in the small intestines do not affect the differentiation of the intestinal epithelial cells but impact the maintenance of ISC, cellular growth in the villi, and epithelial integrity. The balance between stem cell proliferation and differentiation, cell growth, and organ size is regulated by many cellular pathways [98–100]. We performed RNA-seq of a *Drosophila* melanogaster model with dysfunctional peroxisomes exclusively in the intestinal epithelial cells (*Mex* *>* *Pex5-*i) of the midgut, the functional equivalent of mammalian small intestine [101]. Our screen found that the Hippo-Yki pathway is downregulated in *Mex* *>* *Pex5-*i flies compared with control flies. The Hippo (ortholog of Mst1/2 in mammals) pathway was discovered and first described in *Drosophila* and is highly conserved in vertebrates [38–40, 100]. It is a master regulator of tissue growth [102], mainly composed of a phosphorylation cascade that leads to the activation of genes that stimulate cell survival, stem cell maintenance, and proliferation [103]. The Hippo signaling network responds to various environmental cues, including energy stress and metabolic changes [101, 102]. In addition to activating the core Hippo kinases to inhibit YAP (Yki in *Drosophila*), upstream signals can also directly suppress the nuclear localization of YAP/Yki [40, 104, 105]. We confirmed in mice that alteration of peroxisome biogenesis affects cholesterol metabolism, leading to the inhibition of Yap/Yki-signaling in *Drosophila* and mice. Multiple points of crosstalk between lipid metabolism and Hippo signaling have been identified [106]. Here, we defined a mechanism by which p-Yap is sequestered out of the nucleus at cholesterol-rich domains in *Pex2*^*−/−*^ IEC. Cholesterol metabolism is known to be dysregulated in the *Pex2*^*−/−*^ mouse model [86]; particularly, Kovacs et al., 2020 observed a redistribution of cholesterol pools at the subcellular level in mutants. Our experiments confirmed the presence of abnormal cholesterol agglomerates in the cell membrane and cytoplasm using *Pex2*^*−/−*^ MIO-derived monolayers. Moreover, we observed the colocalization of cholesterol and p-Yap, implying that the cholesterol sequesters p-Yap. This interruption in Yap signaling, responsible for cell growth defects and defects in epithelial permeability, is independent of the activation status of the upstream regulator Mst1/2 (or Hpo in *Drosophila*). These findings provide new insights into how metabolism regulates YAP/Yki signaling in relation to IEC metabolism and epithelial integrity.

Controlling the lipid composition of the cell membrane is crucial to regulating junctional protein organization [107, 108]. Whether cholesterol defects inhibit YAP/Yki signaling that then, in turn, affects junctional protein distribution or whether the accumulation of cholesterol affects junctional protein distribution directly is not clear at this point. However, we demonstrated that overexpression of Yki in *Mex* *>* *Pex5-*i enterocytes or depletion of cellular cholesterol in *Pex2*^*−/−*^ intestines or in *Mex* *>* *Pex5-*i enterocytes rescue epithelial structure and barrier function.

Defects in epithelial regeneration, epithelial damage, and excessive intestinal permeability are well-known features of intestinal inflammatory conditions such as celiac disease and IBD [109, 110]; thus, we hypothesized that peroxisome dysfunction and p-Yap localization could be part of the mechanism underlying IBD. We analyzed HIO-derived monolayers from a small cohort of patients with inflammatory diseases of the gastrointestinal tract and healthy donors, and, intriguingly, we found a significant decrease in peroxisome number per cell in patients with severe CD disease. Peroxisome number can increase or decrease in various pathologies [77, 91, 111]; in an earlier report, epithelial cells in human colon carcinomas were found to have a lowered number of peroxisomes compared to healthy controls [112], and more recently, a study showed that peroxisome activity was a useful tool to stratify colorectal cancer patients [113]. Here, we reported that inactive CD patients showed a significant but marginal reduction in peroxisomal numbers in their intestines; however, peroxisomal numbers proportionally decreased with the degree of activation and severity of the disease. Beyond simple numbers of peroxisomes, we report that in cells with dysfunctional peroxisomes and CD patient cells the fraction of nuclear p-Yap was significantly reduced. This pattern of p-Yap sequestration was rescued, and great p-Yap translocated to the nucleus in cells treated with MβCD to reduce cholesterol. Dysregulated p-Yap localization was not observed in cells from non-IBD pathologies. Our work not only describes a novel process whereby peroxisomes influence epithelial integrity but also advances the idea that peroxisomes are novel markers of IBD.

In conclusion, our study shows that peroxisome-mediated cholesterol catabolism is important in regulating the Yap activity, thus identifying a novel mechanism of Yap-signaling conserved from flies to humans and providing new insight into the mechanisms that maintain homeostasis in the intestinal epithelium. The peroxisome-cholesterol-Yap network was not found in another inflammatory disease of the gastrointestinal tract in our study, suggesting that this network could be specific for IBD-type intestinal inflammation, and it could be targeted in novel therapies.

### Supplementary information

Supplementary Figure legends

Supplementary Figure1

Supplementary Figure 2

Supplementary Figure 3

Supplementary Figure 4

Supplementary Figure 5

Supplementary Figure 6

## Data Availability

The authors state that all data necessary to confirm the conclusions are represented in the manuscript.
