# Supplementary material for: Peroxisomal cholesterol metabolism regulates yap-signaling, which maintains intestinal epithelial barrier function and is altered in Crohn’s disease
Source: Cell Death Dis. 2024 Jul 28;15(7):536. doi: 10.1038/s41419-024-06925-x (PMC11284232; doi:10.1038/s41419-024-06925-x)
Supplement: Supplementary file 1 — Supplementary Figure legends [file 41419_2024_6925_MOESM1_ESM.docx]

**Supplementary Legends**

**Extended data Figure 1. Peroxisome dysfunction does not affect the differentiation of intestinal cells**

**A** Imaging of hematoxylin and eosin staining of colonic FFPE sections from day 0 pups. The bar graph represents the value of the epithelial thickness, n=30 slides from 3 mice per genotype. Scalebar=50 µm. **B** Representative TEM images of the colon of the reported genotypes at day 0. Images with an occluded lumen (such as in the *Pex2^-/-^*) were quantified in each genotype, and the number is reported as the percentage of the total number of images; n=3 mice. Scalebar=2 µm. **C** IF image of TUNEL in cryosection of WT and *Pex2^-/-^* colons. The bar graph represents the number of TUNEL-positive spots in the lumen per ROI; n=3 mice. Scalebar=500 µm. **D** RT-qPCR analyses of WT and *Pex2^-/-^* MIO derived from the small intestine to evaluate the expression levels of intestinal lineage markers *Vil1*, *Muc2*, *Lyz1*, and *Chga* for enterocytes, goblet cells, Paneth cells, and enteroendocrine cells, respectively, n=3 MIO culture established from 3 mice. **E** IF image of Ck20 (IEC marker used in monolayers) and Chromogranin A (ChgA), a marker for enteroendocrine cells) in fully differentiated MIO-derived monolayer. **F** IF image of Ki67 in WT and *Pex2^-/-^* fully differentiated MIO-derived monolayer. The bar graph represents the number of Ki67-positive cells per ROI; n=3 mice. Each dot represents the averaged number in10 images. Scalebar=500 µm.

**G** IF image of Claudin7 (Cldn7), **H** E-Cadherin (E-Cad), and **I** Cd49f in cryosections of the small intestine of animals of the reported genotypes. Scalebar=10 µm or Scalebar=20 µm. The bar graph reports the value of MFI of Cldn7 staining in *Pex2^-/-^* cryosection versus the MFI value in WT intestines or the median MFI value of E-cad signal and Cd49f staining WT and *Pex2^-/-^* cryosections. 10 images per mouse were analyzed for each G experiment, n = 3 mice per genotype. In all bar graphs, the error bars represent standard deviations**.** Significance was determined using Student's t-test. ns = not significant; * p<0.05.

**Extended data Figure 2 Dysfunctional peroxisome affects DE-cadherin staining in *Drosophila* midguts. A**, **B** Imaging of Coracle (Cora) and Discs Large 1 (DLG1), in midguts of flies of the reported genotypes Scalebar=5 µm. **C** Imaging of DE-cadherin (DE-cad), in midguts of flies of the reported genotypes. Scalebar=10 µm.  In (**A**), the bar graphs report the MFI of the Cora signal and the membrane thickness. In (**B**) and (**C**), the bar graphs report MFI per ROI. In (**C**), the bar graphs report the number of DE-cad-positive cells through the z-stack and the MFI of the DE-cad-positive cells, respectively. In all the experiments, n=30 guts per genotype. In all bar graphs, the error bars represent standard deviations**.** Significance was determined using Student's t-test. ^∗∗∗∗^p < 0.0001; ^∗∗∗^p < 0.001; ^∗∗^p < 0.01; ^∗^p < 0.05; ns not significant.

**Extended data Figure 3. Original Blot panel 4D.** The panels represent the full-size western blots for A) p-Yap and B) α-Tubulin, as reported in Figure 4, panel D.

**Extended data Figure 4. Original Blot panel 4G.** The panels represent the full-size western blots for A) p-Mst1/2 and B) α-Tubulin, as reported in Figure 4, panel D.

**Extended data Figure 5. Original Blot panel 4H.** The panels represent the full-size western blots for A) p-Hpo and B) α-Tubulin, as reported in Figure 4, panel D.

**Extended data Figure 6 mTor/SREB transcriptional signatures are not detectable in *Pex2^-/-^* small intestines A**  Quantification of relative gene expression in WT and *Pex2^-/-^* small intestines. n=6. **B** Imaging of Filipin III staining in control MIO-derived monolayers treated with increasing doses of MβCD for 2 hours; n=10 images per condition. Scalebar=10 µm.   The bar graphs report the thickness of Filippin-stained membrane in WT MIO-derived monolayers treated with increasing doses of MβCD. **C** The bar graphs report the thickness of the Filippin-stained membrane in WT HIO-derived monolayers of healthy control and patients affected by Macro CD. Significance was determined using Student's t-test.  ^∗∗∗^p < 0.001; ^∗∗∗∗^p < 0.0001; ns not significant.
