# Supplementary figures and images for: Peroxisomal cholesterol metabolism regulates yap-signaling, which maintains intestinal epithelial barrier function and is altered in Crohn’s disease
Source: Cell Death Dis. 2024 Jul 28;15(7):536. doi: 10.1038/s41419-024-06925-x (PMC11284232; doi:10.1038/s41419-024-06925-x)

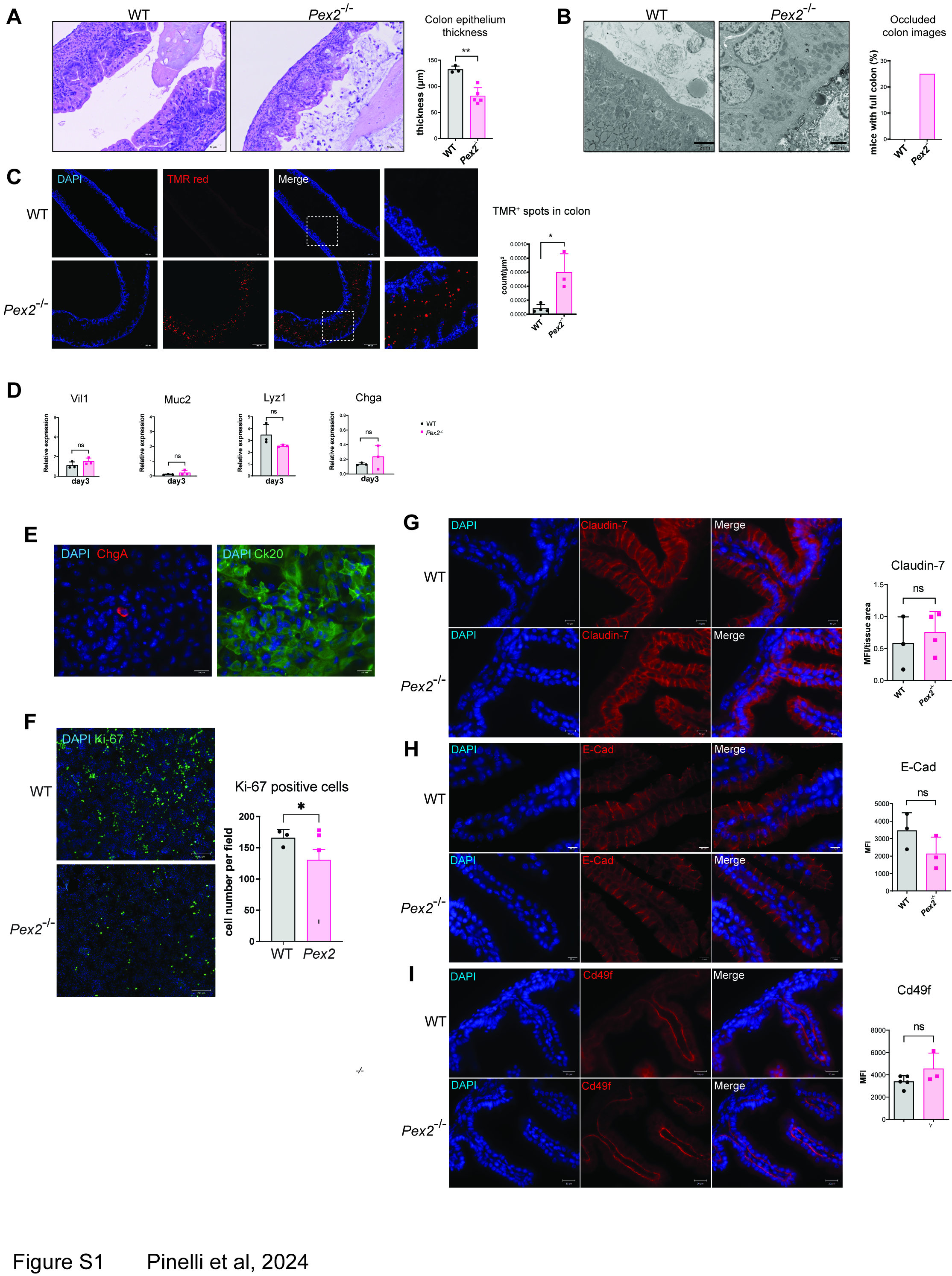

Supplement: Supplementary file 2 — Supplementary Figure1 [file 41419_2024_6925_MOESM2_ESM.tif]

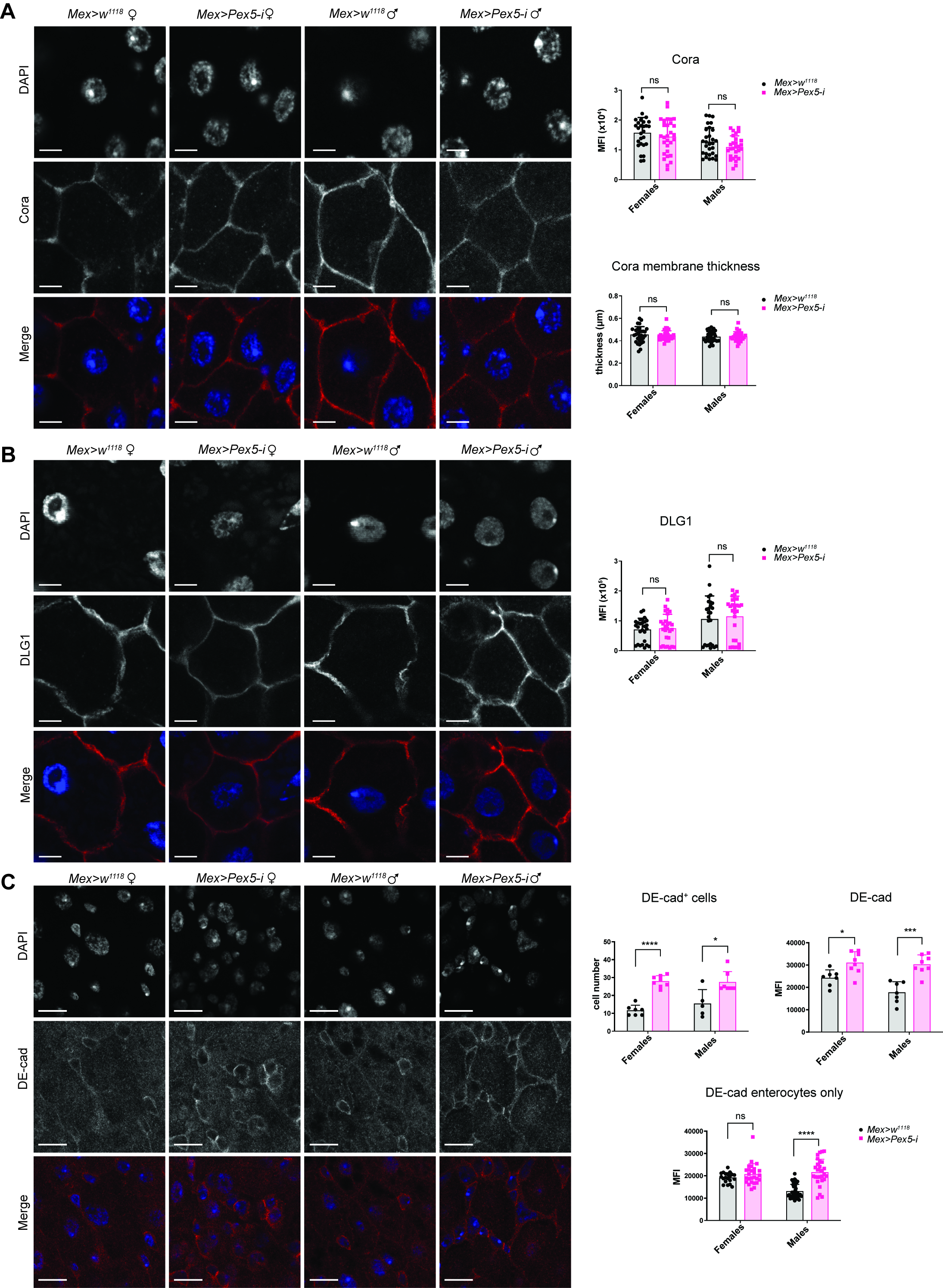

Supplement: Supplementary file 3 — Supplementary Figure 2 [file 41419_2024_6925_MOESM3_ESM.tif]

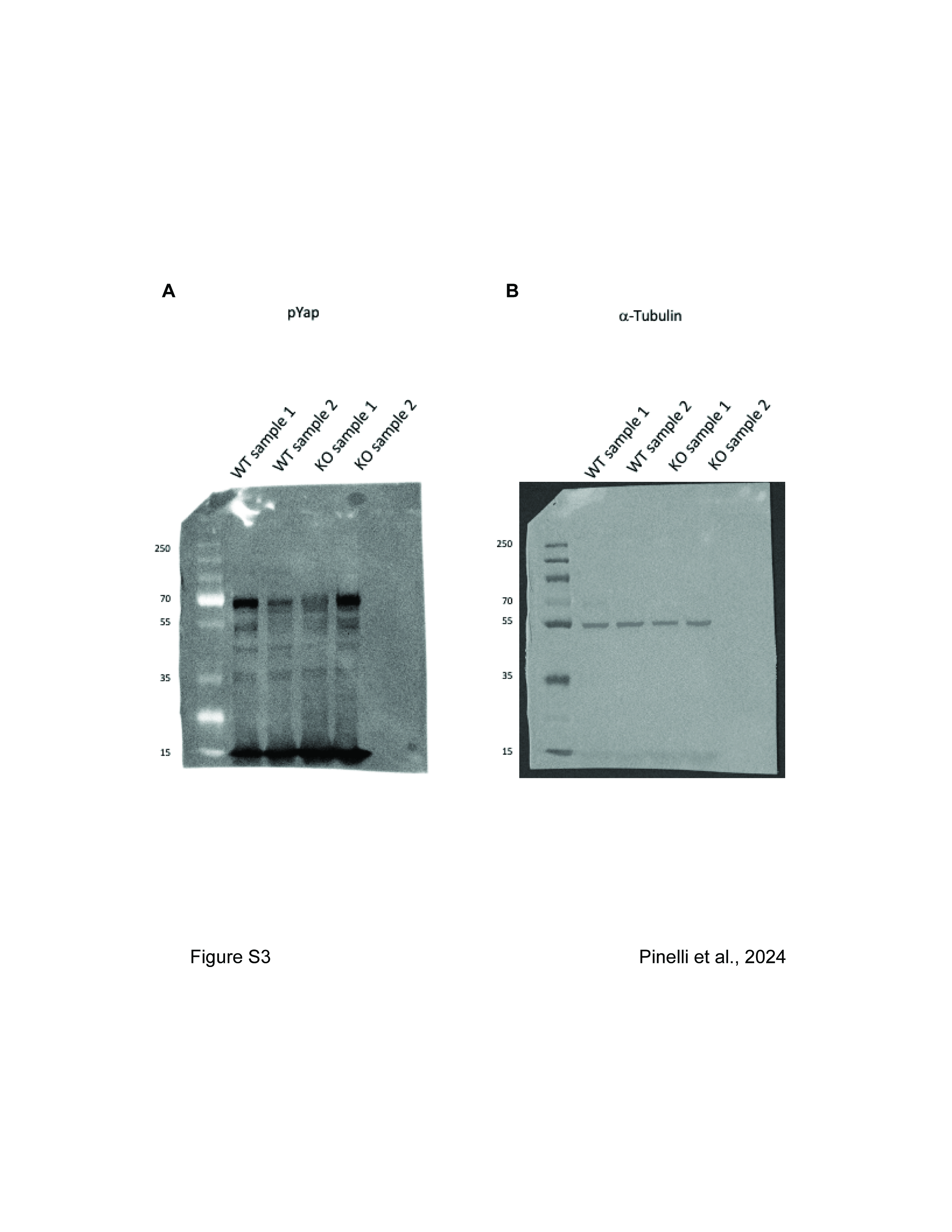

Supplement: Supplementary file 4 — Supplementary Figure 3 [file 41419_2024_6925_MOESM4_ESM.tif]

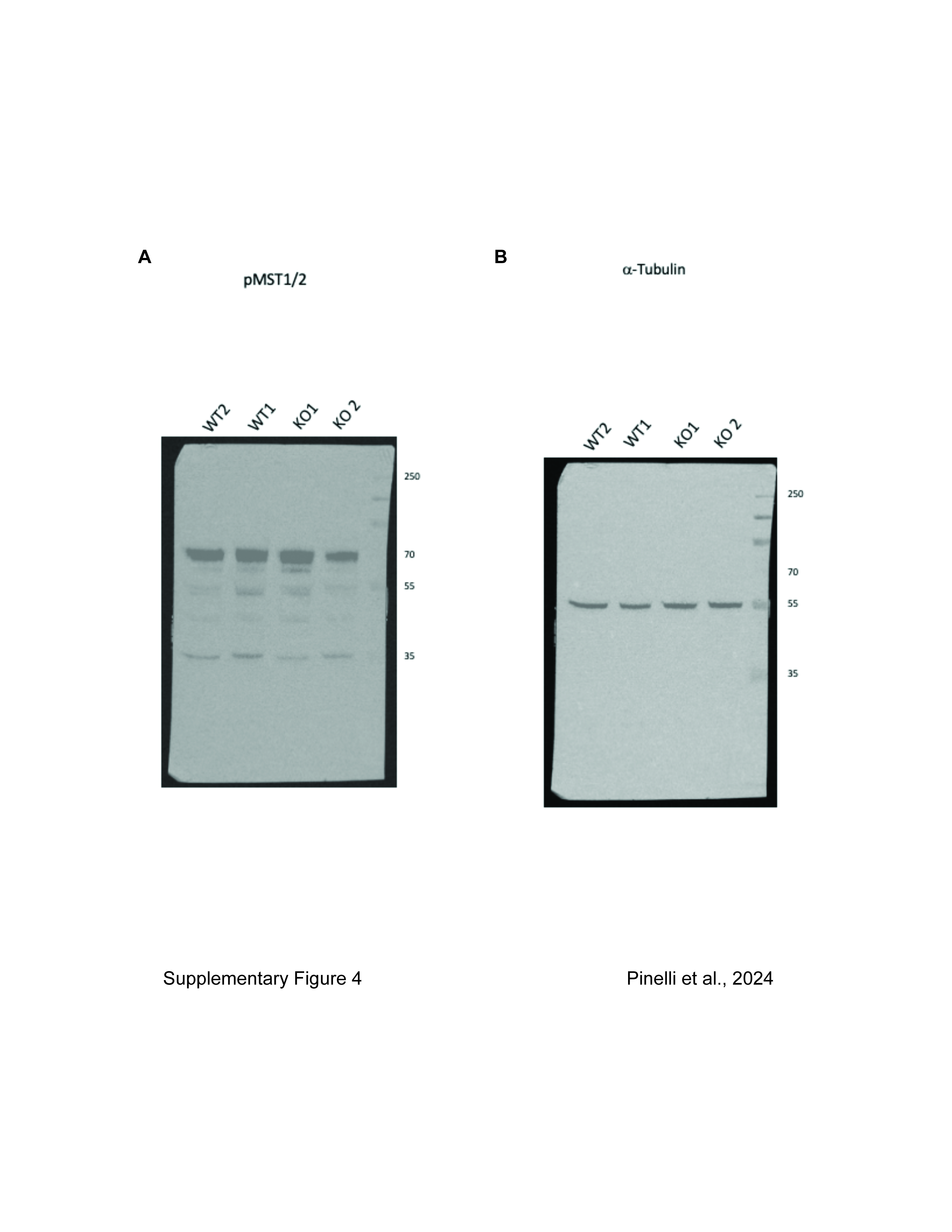

Supplement: Supplementary file 5 — Supplementary Figure 4 [file 41419_2024_6925_MOESM5_ESM.tif]

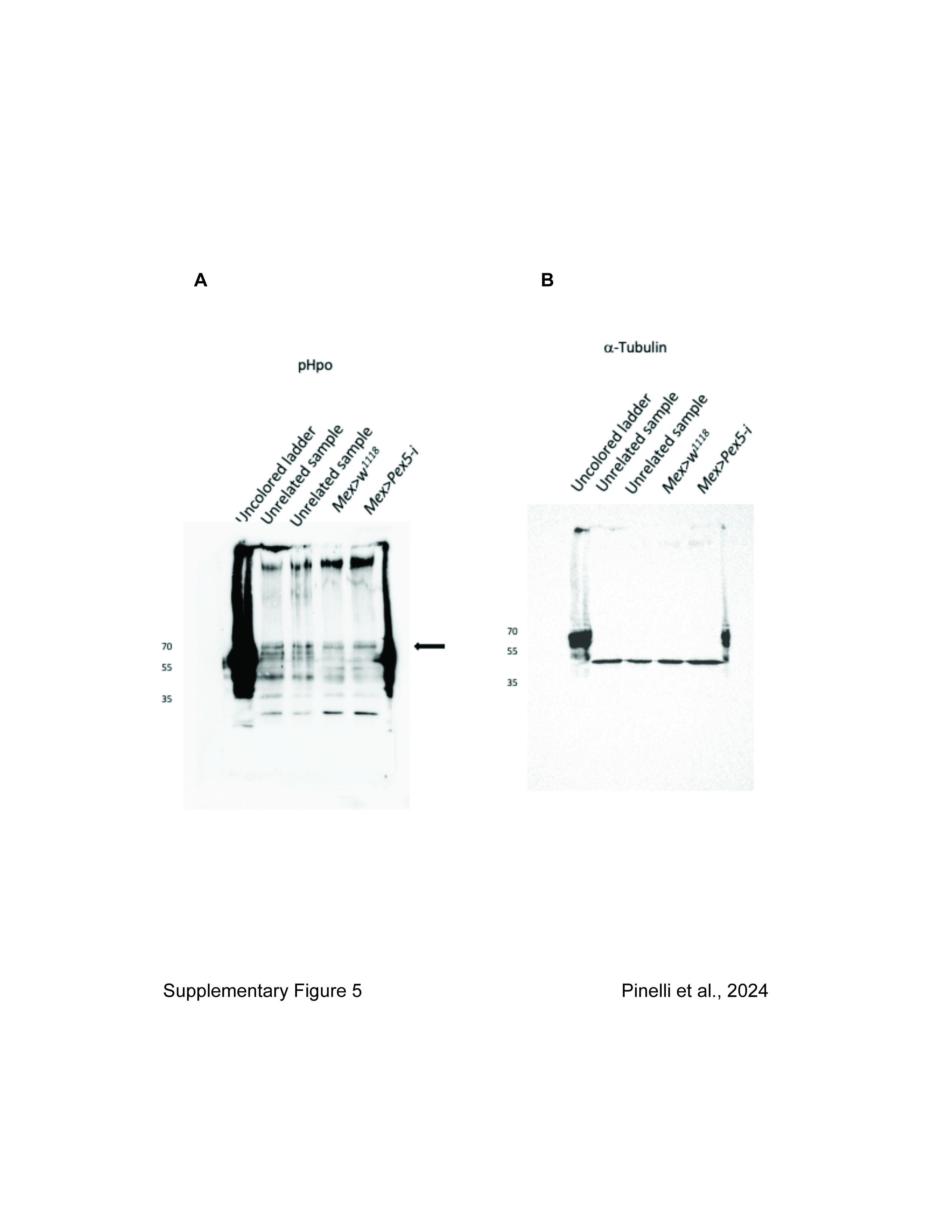

Supplement: Supplementary file 6 — Supplementary Figure 5 [file 41419_2024_6925_MOESM6_ESM.tif]

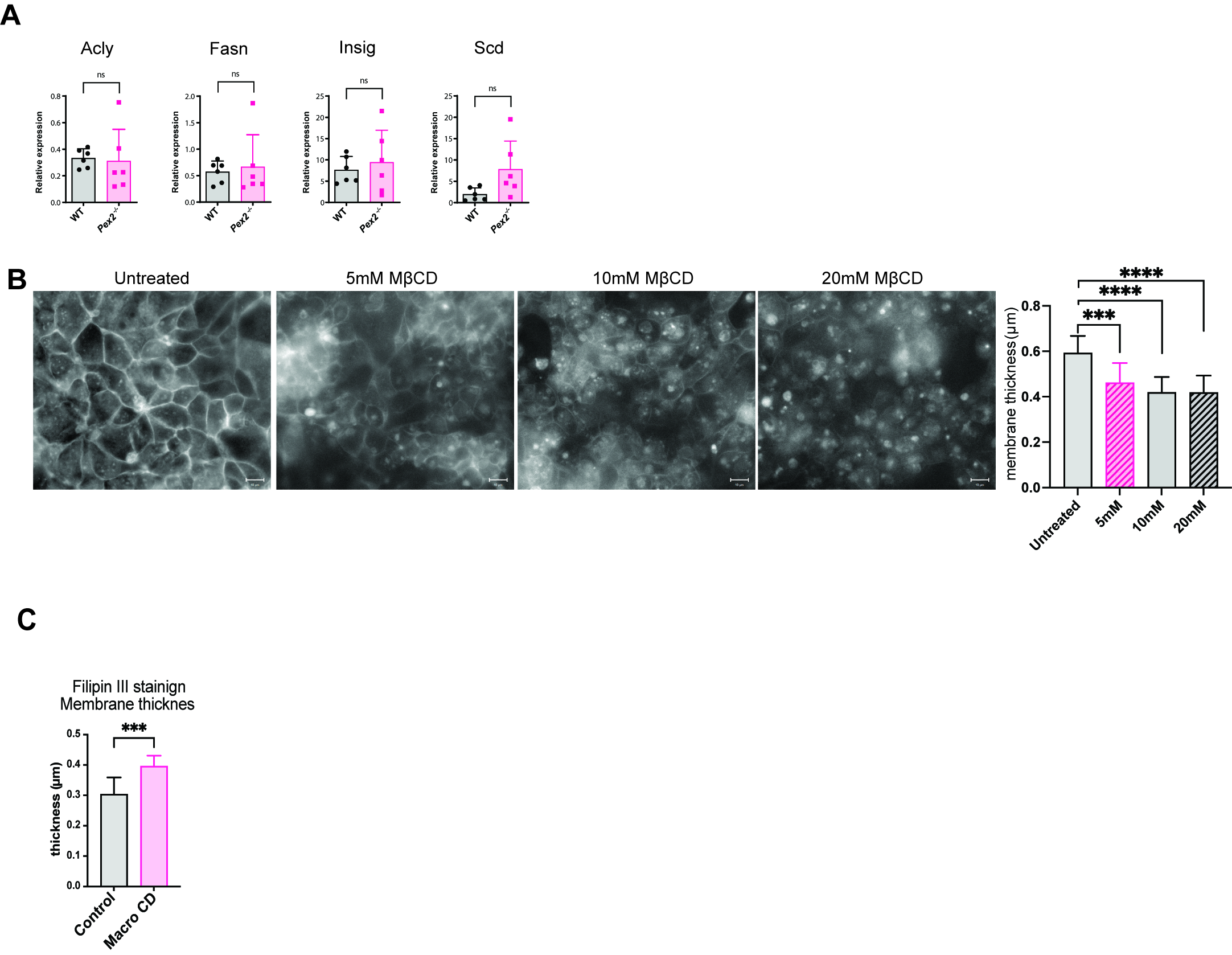

Supplement: Supplementary file 7 — Supplementary Figure 6 [file 41419_2024_6925_MOESM7_ESM.tif]
